# A common cellular response to broad splicing perturbations is characterized by metabolic transcript downregulation driven by the Mdm2–p53 axis

**DOI:** 10.1242/dmm.050356

**Published:** 2024-03-01

**Authors:** Jade E. Varineau, Eliezer Calo

**Affiliations:** ^1^ Department of Biology, Massachusetts Institute of Technology, Cambridge, MA 02139, USA; ^2^ Koch Institute for Integrative Cancer Research, Massachusetts Institute of Technology, Cambridge, MA 02139, USA

**Keywords:** Craniofacial disorders, Splicing, P53

## Abstract

Disruptions in core cellular processes elicit stress responses that drive cell-state changes leading to organismal phenotypes. Perturbations in the splicing machinery cause widespread mis-splicing, resulting in p53-dependent cell-state changes that give rise to cell-type-specific phenotypes and disease. However, a unified framework for how cells respond to splicing perturbations, and how this response manifests itself in nuanced disease phenotypes, has yet to be established. Here, we show that a p53-stabilizing *Mdm2* alternative splicing event and the resulting widespread downregulation of metabolic transcripts are common events that arise in response to various splicing perturbations in both cellular and organismal models. Together, our results classify a common cellular response to splicing perturbations, put forth a new mechanism behind the cell-type-specific phenotypes that arise when splicing is broadly disrupted, and lend insight into the pleiotropic nature of the effects of p53 stabilization in disease.

## Teaser

**Summary:** This work classifies how cells respond when their splicing is broadly disrupted, and explores the relevance of this response to cell-type-specific phenotypes.

## INTRODUCTION

mRNA splicing is a core process in cells, which creates proteomic diversity and can drive changes in cell state (Montes et al., 2019; Scotti and Swanson, 2016). Thus, altering splicing can have widespread effects on health and disease. The impacts of splice site mutations in a single gene generally have more localized effects on splicing, altering the amount, length or composition of the resulting protein, then impacting gene-specific downstream cellular pathways. However, how cells respond when splicing is broadly perturbed – through disruptions of core components in the splicing machinery or use of small molecule splicing inhibitors – is unclear.

Much of what is known about the cellular response to splicing perturbations is derived from the study of spliceosomopathies, developmental disorders caused by haploinsufficiency of a splicing factor (Beauchamp et al., 2020). Some of these disorders primarily impact tissues derived from the neural crest – structures of the lower face and jaw, retinas, limbs, and heart (Park et al., 2022). Individuals with these disorders possess an underdeveloped lower jaw, smaller facial structure and abnormalities of the ear, among other less-penetrant characteristics (Beauchamp et al., 2020). However, a common cellular response to splicing perturbations, which results in specific impacts to the neural crest, has not been fully classified.

Animal models of spliceosomopathies have illuminated our understanding of these disorders but have limitations in their insights into a common cellular response to splicing perturbations. One of the best-studied spliceosomopathies is mandibular facial dysotosis with microcephaly (MFDM), a disorder caused by haploinsufficiency of *EFTUD2,* a highly conserved GTPase in the U5 small nuclear ribonucleoprotein (snRNP) complex (Brenner and Guthrie, 2006, 2005; Fabrizio et al., 1997; Lines et al., 2012). Disease-associated mutations in *EFTUD2* are primarily missense mutations, predicted to result in EFTUD2 loss-of-function (Thomas et al., 2020). When modeling MFDM in zebrafish, fish heterozygous for *eftud2* do not display a phenotype; however, homozygous knock-out of *eftud2* results in an under-formed lower jaw, impaired brain development and widespread apoptosis throughout all regions of the neural crest (Deml et al., 2015; Lei et al., 2017; Wu et al., 2019). Similarly, mouse models of MFDM do not recapitulate the haploinsufficiency of *Eftud2*, and a neural-crest-specific double KO of *Eftud2* results in widespread apoptosis and a partially p53-dependent craniofacial phenotype (Beauchamp et al., 2022, 2021). These models highlight the importance of the neural crest as a primary cell type impacted by splicing perturbations. However, phenotypes observed in animal models are much more severe compared with those observed in affected individuals, thereby providing limited insight into the cellular alterations that occur when splicing is perturbed to a more-moderate level and relevant to human disease.

In addition to the neural crest, other cell types are sensitive to broad splicing perturbations. The use of splicing inhibitors in conjunction with more-typical therapeutic agents shows promise as an effective cancer treatment (Lee and Abdel-Wahab, 2016; Salton and Misteli, 2016; Zhang et al., 2019). Thus, it seems as though highly proliferative, migratory, multipotent cell types – like the neural crest or cancer cells – are uniquely sensitive to splicing perturbations. Identifying the cell-state changes that occur when splicing is broadly disrupted will illuminate the mechanism behind the sensitivity to splicing perturbations in these cell types.

The cellular response to broad splicing perturbations is mediated through the tumor protein p53 by a mechanism that has yet to be fully elucidated. In cells, knockdown (KD) of splicing factors and chemical inhibition of the spliceosome by small molecules results in p53 stabilization (Allende-Vega et al., 2013; Huang et al., 2018), and neural crest cells have a unique heightened sensitivity to p53 stabilization (Bowen et al., 2019; Calo et al., 2018). Despite this knowledge, a unifying link between splicing perturbations in general and stabilization of p53 has yet to be classified. Furthermore, the craniofacial phenotypes that arise from aberrant p53 stabilization are, to a certain extent, independent of caspase-9 and PUMA (officially known as BBC3), factors involved in the p53-mediated apoptosis pathway (Bowen et al., 2021). This suggests that p53 mediates its cell-type-specific impacts through a pathway other than canonical p53-mediated apoptosis and there may be other alternative cellular responses or altered cell death pathways at play when splicing is perturbed.

Here, we used mouse embryonic stem cells (mESCs) to understand the cellular response to moderate splicing perturbations. We classified an *Mdm2* alternative splicing event as a common event that arises in response to multiple splicing perturbations and directly links broad splicing disruption to p53 stabilization. Additionally, we found that splicing perturbations drive p53-dependent downregulation of glycolytic transcripts. Then, we expanded the study of splicing perturbations and metabolic alterations to a zebrafish developmental model. We demonstrated that, as in mESCs, *mdm2* is alternatively spliced in zebrafish and glycolytic transcripts are downregulated in response to widespread splicing perturbations. We also showed that craniofacial phenotypes resulting from inhibition of glycolysis phenocopy those of small-molecule splicing inhibition. Finally, we examined the downregulation of glycolytic transcripts in other contexts of p53 stabilization and propose this cell-state change as a chronic cellular response to prolonged p53 stabilization with relevance to disease phenotypes. All in all, our results define a common cellular response to splicing perturbations, and lend insight into the cell-type-specific impacts of this response.

## RESULTS

### *Eftud2* deficiency has dosage-dependent effects on alternative splicing of *Mdm2* in mESCs

To better understand the alternative splicing events and transcriptional cell-state changes that occur in response to splicing perturbations, we first focused specifically on the impact *Eftud2* deficiency has on mESCs. For this study, we chose to use mESCs grown in defined medium conditions using N2B27 medium supplemented with leukemia inhibitory factor (LIF) and additional kinase inhibitors as previously described (Thomson et al., 2011; Ying et al., 2008; Ying and Smith, 2003). These mESCs are highly proliferative and exhibit a pluripotent state, characteristics similar to those of neural crest cells (Bronner and LeDouarin, 2012; Ying and Smith, 2003). However, unlike neural crest cells, mESCs are less prone to apoptosis, allowing us to probe cell-state changes that occur in addition to, or instead of, apoptosis in response to broad splicing perturbations (Aladjem et al., 1998; Ayaz et al., 2022; Calo et al., 2018; Raj et al., 2022). This is an important consideration as apoptosis-independent mechanisms have been proposed to contribute to developmental phenotypes associated with splicing perturbations (Bowen et al., 2021), but these mechanisms have been difficult to study in animal models of spliceosomopathies.

**Fig. 1.**
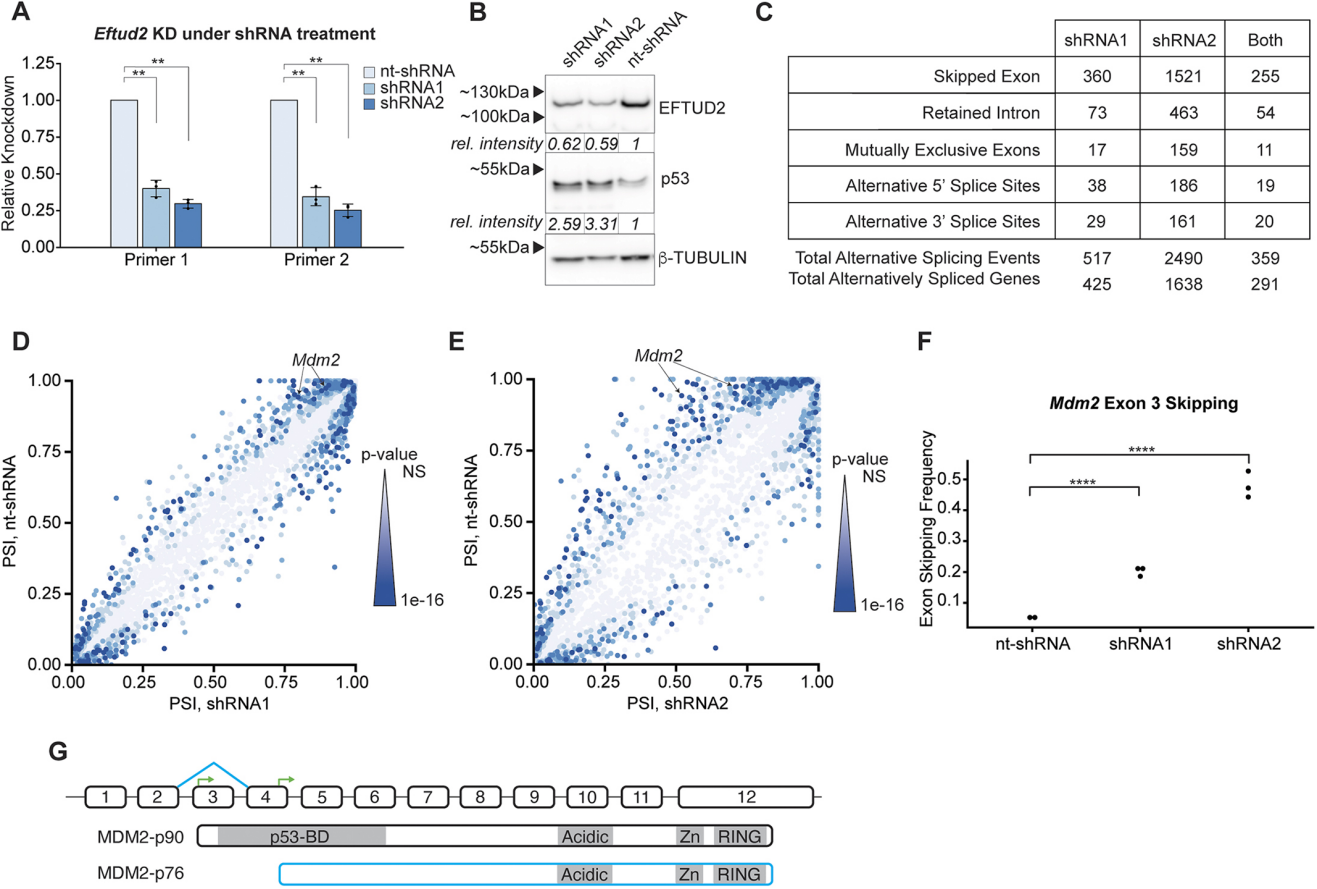
**Dosage-dependent alternative splicing of Mdm2 following *Eftud2* KD.** (A) Plotted are levels of knocked down *Eftud2* (*Eftud2* KD) as assayed by qPCR for two independent primer pairs for Eftud2, after using two independent *Eftud2-*targeting shRNAs (shRNA1 or shRNA2) or nt-shRNA as control. Bar graphs show 2^−ΔΔCt^, representing *Eftud2* KD compared to *Eftud2* levels after control treatment. Each biological replicate of shRNA KD was compaired pairwise to the corresponding nt-shRNA biological replicate to calculate KD levels, and each biological replicate is the average of three technical replicates. Error bars represent ±standard deviation of biological replicates. Significance values were determined using Student's *t*-test on paired ΔCt values. ΔCt values were calculated by normalizing raw values to mitochondrial rRNA levels. **P<0.01. (B) Western blots of mESC lysates, showing levels of EFTUD2 and p53 after treatment with shRNA1 or shRNA2 to knock down *Eftud2* or with control nt-shRNA; levels of β-tubulin were used as loading control. Relative intensity values, standardized to those of β-tubulin and normalized to nt-shRNA, are listed below each protein band for both EFTUD2 and p53. (C) Summary of significant [false discovery rate (FDR)<0.01] alternative splicing events observed in response to shRNAs targeting *Eftud2,* calculated by using the rMATS tool. (D,E) Plotted are percent-spliced-in (PSI) of skipped-exon events detected by rMATs in mESCs treated with either shRNA1 (D) or shRNA1 (E) (*x*-axis) to knock down *Eftud2*, or with control nt-shRNA (*y*-axis). Plotted points represent skipped-exon events present in response to either KD, with a higher number of skipped-exon events in shRNA2 than in shRNA1. Darker colors indicate more-significant *P*-values, NS, not significant. Both *Mdm2* skipped-exon events were skipping Exon 3, defined as separate events by rMATS due to slight differences in the upstream exon. (F) Exon-skipping frequency ((defined as 1 minus the percent-spliced-in value calculated by rMATS) of *Mdm2* Exon 3 skipping event in mESCs treated with shRNA1 or shRNA2 to knock down *Eftud2*, or with nt-shRNA (control). Significance values were determined by rMATS with a likelihood-ratio test. ****P<0.0001. (G) Schematic of alternative *Mdm2* splicing events of interest (top) and their resulting protein products MDM2-p90 and MDM2-p76 (middle and bottom, respectively). Numbered boxes indicate exons. Blue diagonal lines connecting exons 2 and 4 indicate the exon-skipping event. Green bent arrows indicate start codons. Skipping of Exon 3 gives rise to an MDM2 isoform (i.e. MDM2-p76) that lacks the complete p53-binding domain. p53-BD, p53-binding domain; Acidic, acidic domain; Zn, Zinc finger domain; RING, really interesting new gene (RING) finger domain. Drawings are not to scale.

We reasoned it would be important to study the cell-state changes that occur in response to weaker splicing perturbations, putatively more closely mimicking the haploinsufficient cellular response to splicing perturbations as it occurs in MFDM in affected individuals. Thus, we knocked down *Eftud2* with two independent small hairpin RNAs (shRNA1 or shRNA2). shRNA1 targets the 3′ UTR and has a 55–68% KD efficiency, as determined by qPCR. shRNA2 targets the coding sequence and has a 64–79% KD efficiency as determined by qPCR (Fig. 1A) (KD efficiency calculated by 2^−ΔΔCt^, see qPCR and sqRT-PCR in Materials and Methods). Use of either shRNA results in reduced levels of EFTUD2, as measured by western blotting (Fig. 1B). Consistent with previous literature, we observed higher p53 protein levels in response to shRNA treatment when compared to a non-targeting shRNA (nt-shRNA) as control (Fig. 1B) (Beauchamp et al., 2021; Wu et al., 2019). Using two independent shRNAs strongly argues that any common changes observed after KD are due to *Eftud2* deficiency, thereby allowing us to observe whether any alterations correlate with the KD level.

We transiently infected mESCs with either of the two *Eftud2-*targeting shRNAs or control nt-shRNA using lentiviral infection and collected the infected cells after three days of selection. Then, we performed RNA sequencing (RNA-Seq) on the infected mESCs using three biological replicates for each targeting shRNA and two biological replicates for the nt-shRNA. RNA-Seq read mapping was carried out to maximize the number of splice junction reads to capture any splicing alterations that occur in response to *Eftud2* KD (see RNA sequencing and analysis in Materials and Methods). We then analyzed alternative splicing by using the rMATS computational tool (Shen et al., 2014).

As expected, we detected altered splicing in a wide variety of transcripts in response to *Eftud2* KD. Both *Eftud2* shRNAs resulted in widespread mis-splicing, with skipped exons the most common form of alternative splicing (Fig. 1C). We observed more-significant [false discovery rate (FDR)<0.01] alternative splicing events with shRNA2 than shRNA1, which is consistent with the higher level of *Eftud2* KD in response to using shRNA2 (Fig. 1A-C). Gene Ontology (GO) over-representation analysis for biological processes shows that alternatively-spliced genes were enriched for terms relating to RNA splicing and stability (Fig. S1A-D), consistent with previous findings for splicing factor perturbations (Alam et al., 2022; Wood et al., 2019; Wu et al., 2018).

As *Eftud2* deficiency has been associated with the activation of p53, we analyzed our alternative splicing analysis for splicing events that could link *Eftud2* KD to p53 activation. Gene ontology analysis demonstrated that there is no gene-set or pathway over-represented among alternative splicing events that immediately point to p53 (Fig. S1A-D). We then turned our focus to the significant exon skipping events in more detail. Among the set of highly significant exon skipping events, exon skipping events within *Mdm2* immediately stood out, as MDM2 is a negative regulator of p53. We found that the alternative splicing events in *Mdm2* demonstrate a marked increase compared with those after treatment with control shRNA, are highly significant and correlate with shRNA potency (Fig. 1D-F).

The alternatively spliced exon within *Mdm2*, i.e. Exon 3, contains the primary start codon of MDM2 as well as parts of the p53-binding domain. Thus, increased exon skipping results in an in-frame, N-terminally truncated MDM2 protein that uses an alternative downstream start codon and cannot bind p53 (Fig. 1G) (Cheng and Cohen, 2007; Giglio et al., 2010; Perry et al., 2000; Saucedo et al., 1999). Increases in the ratio of this alternative MDM2 isoform to full-length MDM2 reduce the number of MDM2–MDM4 heterodimers capable of targeting p53 for degradation, thereby stabilizing p53 (Giglio et al., 2010). We also detected an additional alternative splicing event in *Mdm2*, with altered ratio of Exon 4 skipping in response to *Eftud2* KD, which also is predicted to produce a non-p53-binding isoform (Fig. S2A-D). The *Mdm2* Exon 3 mis-splicing event is consistent with previously reported events of *Eftud2* splicing perturbation (Beauchamp et al., 2021) and, intriguingly, splicing perturbations of other genes (Alam et al., 2022; McElderry et al., 2019; Van Alstyne et al., 2018). Our results confirmed a *Mdm2* alternative splicing event that arises from *Eftud2* deficiency in a manner that correlates with its level of knock-down, and provides a direct link between splicing perturbations and aberrant p53 stabilization in response to *Eftud2* deficiency.

### Alternative splicing of *Mdm2* is a common event following broad splicing perturbations in mESCs

Next, we set out to investigate whether *Mdm2* alternative splicing is a common feature of splicing perturbations in mESCs. If so, this could provide a unified mechanism by which broad splicing perturbations result in p53 stabilization and downstream p53-dependent phenotypes.

**Fig. 2.**
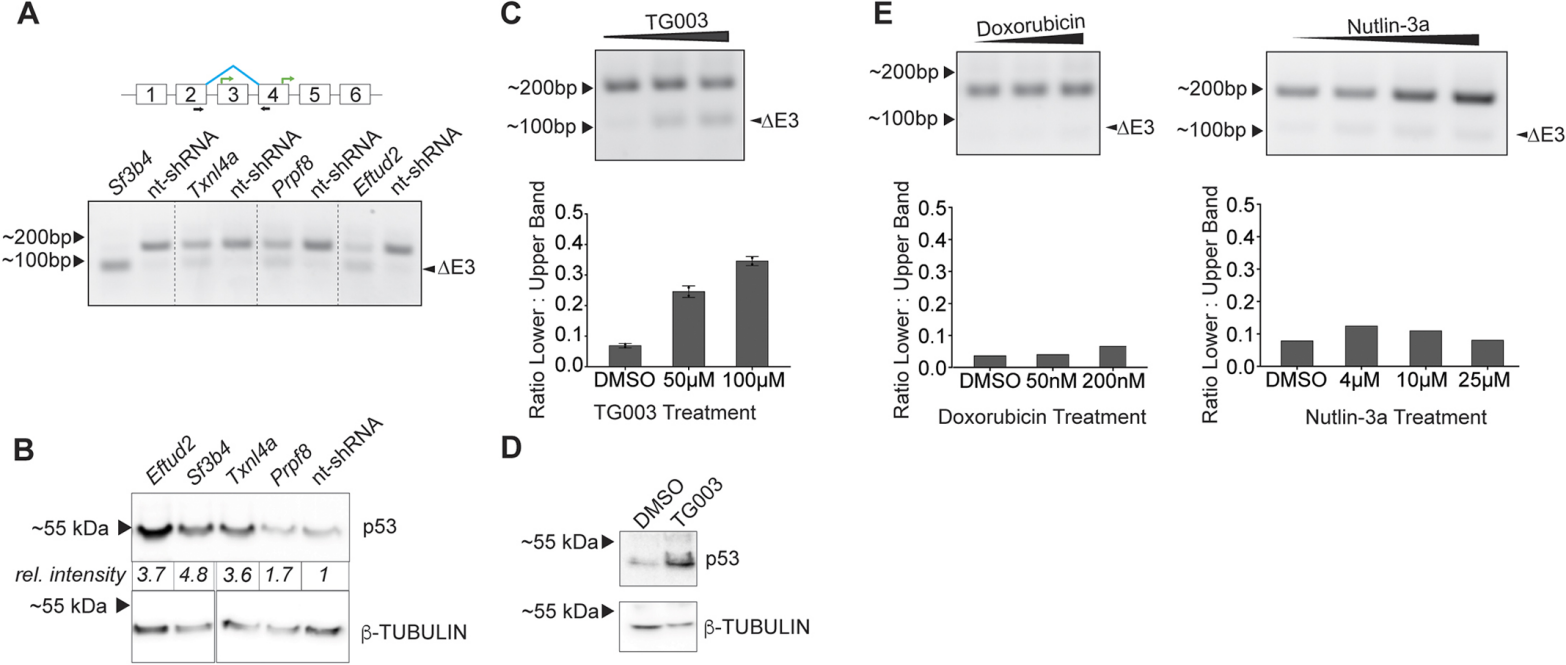
**Mdm2 alternative splicing occurs in response to various splicing perturbations.** (A) Top: Schematic of the region surrounding the *Mdm2* alternative splicing event. Black arrows represent primer binding sites. Green bent arrows represent start codons. Blue diagonal lines connecting exons 2 and 4 indicate exon-skipping events. Bottom: Agarose gel of semi-quantitative reverse transcription PCR (sqRT-PCR) to analyze alternative splicing of *Mdm2* in response to individual shRNA knockdown (KD) of splicing factors Sf3b4, Txnl4a or Prpf8. Approximate size of sqRT-PCR products is as indicated (∼200 bp, ∼100 bp). ΔE3 indicates the product resulting from *Mdm2* exon 3 skipping. (B) Western blotting of mESC lysates. mESCs were infected with different shRNAs specifically targeting splicing factors *Eftud2*, *Sf3b4*, *Txnl4a* or *Prpf8*, or with nt-shRNA (control) as indicated. Bands show levels of p53 (top); β-tubulin (bottom) was used as loading control. Relative intensity (*rel. intensity*) values standardized to β-tubulin and normalized to nt-shRNA are provided under each band. (C) Representative sqRT-PCR (top) of *Mdm2* alternative splicing in response to 4 h of TG003 treatment, and corresponding quantification of sqRT-PCR band intensities (bottom). Error bars represent  ±standard deviation of two biological replicates. (D) Western blot of p53 in response to TG003 treatment; β-tubulin was used as loading control. (E) sqRT-PCR of *Mdm2* alternatively spliced in response to 4 h-treatment with either Doxorubicin (left panels) or Nutlin-3a (right panels). The corresponding quantification of *Mdm2* sqRT-PCR band intensities is plotted below.

To test this, we knocked down three additional splicing factors in mESCs, which have varying roles in the spliceosome: the U5 snRNP component *Txnl4a*, whose haploinsufficiency results in Burn–McKeown syndrome (Simeoni et al., 2005; Wieczorek et al., 2014); *Prpf8*, also a component of the U5 snRNP, essential for the catalytic step of splicing and linked to retinitis pigmentosa (Arzalluz-Luque et al., 2021; Galej et al., 2013; Grainger and Beggs, 2005); and *Sf3b4,* a core subunit of the U2 and U12 snRNPs, which is mutated in Nager syndrome (Devotta et al., 2016; Golas et al., 2003; Yamada et al., 2020). Analysis of semi-quantitative reverse transcription PCR (sqRT-PCR) showed that – like KD of *Eftud2* and compared to control treatment using nt-shRNA – specific KD of any the above splicing factors also resulted in increased presence of the Exon 3-skipping *Mdm2* transcript (Fig. 2A, see ΔE3). We also observed the expected increase in p53 levels when any of the above splicing factors was knocked down with shRNA (Fig. 2B). These results suggest that *Mdm2* alternative splicing and p53 stabilization arise even when disrupting different steps and components of splicing.

To understand whether *Mdm2* alternative splicing occurs in mESCs when splicing is more broadly inhibited, we made use of the potent Clk1 and Clk4 inhibitor TG003 that inhibits the phosphorylation and activation of serine/arginine (SR)-rich proteins, splicing factors involved in the regulation of many steps of splicing, thereby widely perturbing it (Allende-Vega et al., 2013; Ghosh and Adams, 2011; Lebecque et al., 2022; Muraki et al., 2004). To ascertain whether this widespread splicing inhibition induces *Mdm2* alternative splicing, we measured *Mdm2* transcript levels with sqRT-PCR. Indeed, we saw increased alternative splicing of *Mdm2* after treatment with TG003 in a concentration-dependent manner, resulting in stabilization of p53 (Fig. 2C,D). This ability of TG003 to induce *Mdm2* alternative splicing, in a concentration-dependent manner, demonstrates that *Mdm2* alternative splicing is a direct response to broad splicing perturbations.

*Mdm2* is part of a feedback loop with p53 because *Mdm2* is transcriptionally regulated by p53 and the p53 downstream effector ZMAT3 promotes this *Mdm2* alternative splicing event (Bieging-Rolett et al., 2020). As such, we set out to confirm that alternative splicing of *Mdm2* we observed was not due to p53 stabilization but due to broad splicing perturbations. We found that indirect activation of p53 through the DNA-damaging agent Doxorubicin or direct activation of p53 through the MDM2 inhibitor Nutlin-3a did not induce alternative splicing of *Mdm2* (Fig. 2E), confirming that the *Mdm2* alternative splicing event is selective to broad splicing perturbations, and is not downstream of p53 stabilization, in mESCs.

We further pursued the relationship between the p53-mediated regulation of *Mdm2* and alternative splicing by creating p53-null mESC cell lines using CRISPR-Cas9. We observed reduced *Mdm2* alternative splicing in a p53-null background in response to splicing perturbations induced by TG003 treatment or *Eftud2* KD (Fig. S3A,B). This suggests that, although some of the observed *Mdm2* alternative splicing is consistent with a p53 positive feedback loop, the initial alternative splicing event is not due to p53 stabilization but directly caused by broad splicing perturbations. Taken together, our results position *Mdm2* alternative splicing as an upstream event in the ability of cells to trigger a p53-dependent stress response to broad splicing perturbations in p53-expressing cells. In addition, these data point to a model, in which *Mdm2* alternative splicing is triggered by splicing disruption, serving as a sensor for the subsequent cellular response mediated through p53.

### *Eftud2* deficiency results in p53-dependent downregulation of metabolic transcripts

Previous studies have established that broad splicing perturbations result in p53-dependent apoptosis of the neural crest cells (Beauchamp et al., 2021; Lei et al., 2017). However, these experiments modeling splicing perturbations have been conducted in homozygous mutant animals, and genetic experiments in mice have shown that abrogation of p53-mediated apoptosis still results in craniofacial malformations (Bowen et al., 2021). This suggests that other mechanisms are at play in the cellular response to haploinsufficiency of splicing components. To explore what pathways these additional mechanisms could encompass, we strove to identify cell-state changes that occur in cells undergoing splicing perturbations.

We used the RNA-Seq data of *Eftud2* KD mESCs to analyze differential gene expression. Consistent with the KD efficiencies of the shRNAs, we found that, compared to control nt-shRNA, levels of differentially expressed genes were more significant following treatment with shRNA2 than shRNA1 (Fig. S4A). In addition, principal component analysis showed that differential gene expression of biological replicates does cluster according to the potency of the shRNA (Fig. S4B,C).

**Fig. 3.**
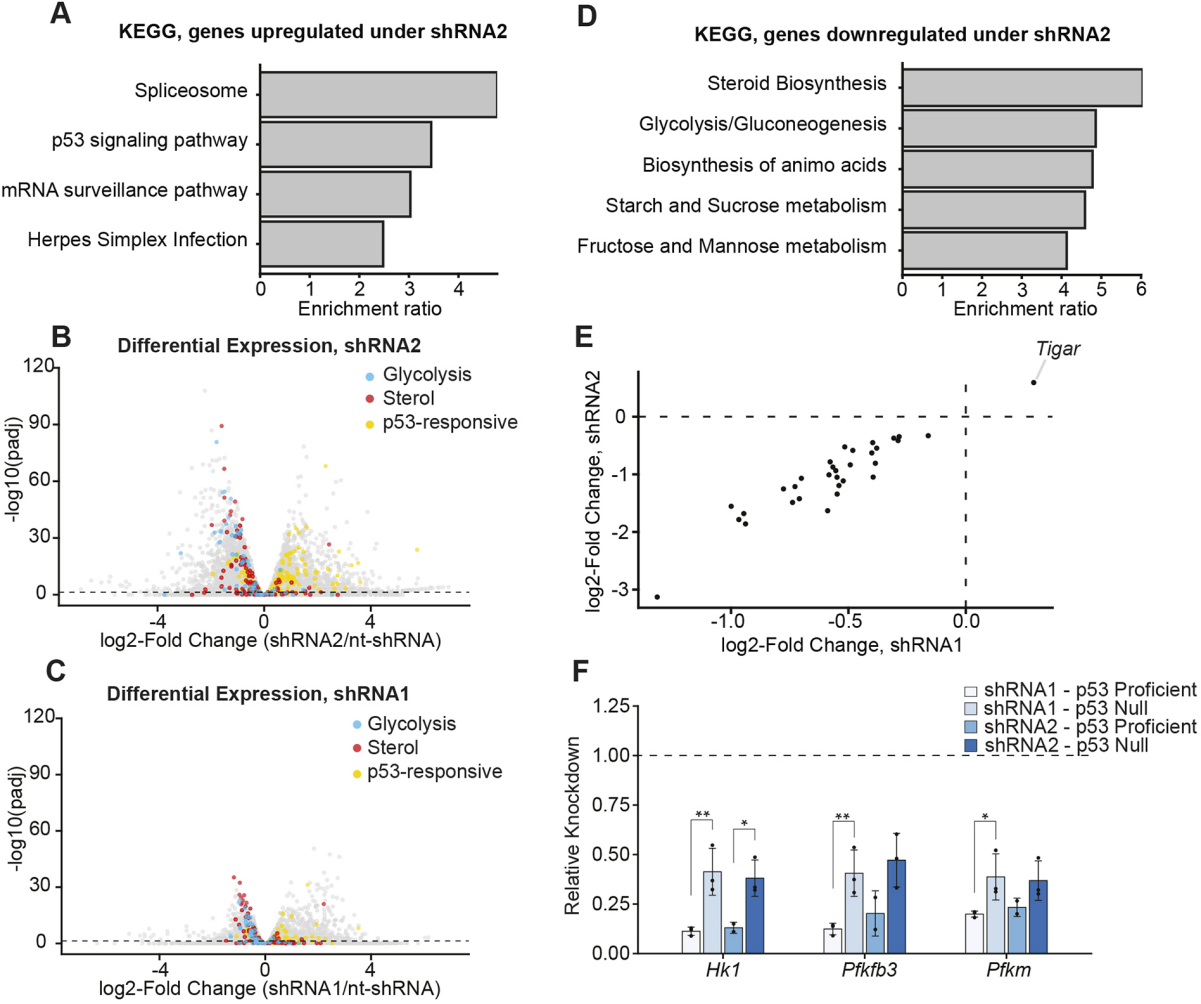
**RNA-seq reveals metabolic transcript downregulation in *Eftud2* KD mESCs.** (A,D) Over-representation analysis of 1000 Kyoto Encyclopedia of Genes and Genomes (KEGG) pathway genes, comparing those most significantly upregulated (A) or downregulated (D) under shRNA2 knockdown **(**KD) and under nt-shRNA. (B,C) Differential expression of all genes analyzed in A and D, comparing the effect of shRNA2 (B) or of shRNA1 (C) with that of nt-shRNA as assayed by DESeq2. Blue, genes involved in hexose sugar metabolism (Glycolysis); red, genes involved in sterol biosynthesis (Sterol); yellow, p53 transcriptional targets (p53 responsive). Components of gene sets and their compilation are described under Compilation of gene set lists in Materials and Methods. (E) Plotted is the log2-fold change of all glycolytic genes significantly differentially expressed in under both shRNA1 and shRNA2. (F) Quantification of qPCR analysis. Plotted is the downregulation of select glycolytic gene transcripts as indicated, following *Eftud2* KD in p53-expressing and p53-null cell lines. Bar graphs show 2^−ΔΔCt^, representing KD relative to control nt-shRNA in the given p53 status (denoted by the dotted line). Each biological replicate of shRNA KD was compaired pairwise to the corresponding nt-shRNA biological replicate to calculate KD levels, and each biological replicate is the average of three technical replicates. Error bars represent  ±standard deviation of biological replicates. Significance values determined by Student's *t*-test on ΔΔCt values. ΔCt values calculated by normalizing raw values to mitochondrial rRNA levels. *P<0.05, **P<0.01.

As animal models of MFDM and other splicing perturbations have demonstrated activation of the p53 pathway (Beauchamp et al., 2021, 2022; Lei et al., 2017; McElderry et al., 2019), we examined the canonical p53 response in *Eftud2* KD cells. Indeed, analysis of Kyoto Encyclopedia of Genes and Genomes (KEGG) pathway genes upregulated upon the more-potent *Eftud2* KD (shRNA2) demonstrated enrichment of the p53 signaling pathway, consistent with activation of the p53 pathway in response to splicing perturbations as previously described (Fig. 3A) (Beauchamp et al., 2022, 2021; Lei et al., 2017). The heightened p53 response was also visible for differential expression of canonical p53-transcriptional targets, as we observed slight upregulation of canonical p53 targets with shRNA2 (Fig. 3B, Figs S4A, S6A). However, although p53 is stabilized after KD in response to either shRNA1 or shRNA2 (Fig. 1B), the canonical p53 transcriptional response was not detected in response to KD in response to shRNA1 (Fig. 3C, Figs S4A, S5A-C, S6B). Thus, we set out to investigate other possible mechanisms through which p53 stabilization could affect a cell-state change after *Eftud2* KD in response to either shRNA.

KEGG pathway analysis of genes downregulated upon *Eftud2* KD revealed transcript enrichment of genes involved in hexose sugar metabolism or sterol biosynthesis (Fig. 3D, Fig. S5D,E). Furthermore, Gene Ontology analysis showed that downregulated genes are enriched in a wide array of metabolism-related terms pertaining to sterol biosynthesis, sugar metabolism, nucleotide metabolism and alcohol metabolism (Fig. S5F). These metabolic signatures were observed after KD of *Eftud2* with the weaker shRNA1, as well as after KD with the stronger shRNA2 (Fig. 3B-D, Fig. S5D-F). The commonality of this transcriptional signature emphasizes that it is the principal cell-state change in response to *Eftud2* deficiency in mESCs. These metabolic signatures have not been previously reported when modeling broad splicing perturbations.

We then further examined alterations in metabolic transcripts after *Eftud2* KD by compiling all genes associated with given metabolic categories and examining their differential expression. This analysis revealed widespread downregulation of the vast majority (87–91%) of transcripts associated with sterol and glucose metabolism, in response to both stronger and weaker KD of *Eftud2* (Fig. 3B,C, Fig. S4A, S6C-F). Differential expression was increased after KD using shRNA2, consistent with the increased level of KD (Fig. 3E, Fig. S6E,F). Very few of the downregulated metabolic transcripts also contained a significant alternative splicing event (Fig. S6G,H). Thus, most of the downregulation observed is due to indirect impacts of splicing perturbations.

Among the alterations regarding metabolic transcript levels, the downregulation of glycolytic transcripts is particularly striking, due to the importance of glycolysis in cell-state transitions in the neural crest and other developmental lineages. To properly undergo epithelial-to-mesenchymal-transition (EMT) the neural crest requires increased glycolysis, and inhibition of glycolysis results in migratory defects (Bhattacharya et al., 2020; Figueiredo et al., 2017). As such, the downregulation of glycolytic transcripts we observed could explain cell-type-specific phenotypes seen in response to *Eftud2* deficiency.

As phenotypes observed in response to *Eftud2* deficiency are partially p53-dependent, we reasoned that downregulation of glycolytic transcripts could be one such cell-state change that results from p53 stabilization. We observed upregulation of *Tigar*, a p53-activated fructose-2,6-bisphosphatase, which negatively regulates glycolysis (Bensaad et al., 2006) (Fig. 3E). Furthermore, p53 is known to regulate glycolysis and glycolytic transcripts through a myriad of other mechanisms (Liu et al., 2019). To test the relationship between glycolytic transcript downregulation and p53, we knocked down *Eftud2* in a p53-null background and performed qPCR on select glycolytic transcripts. Indeed, we observed that, after *Eftud2* KD, downregulation of select glycolytic transcripts was reduced in a p53-null background (Fig. 3F). This finding supports the model that downregulation of glycolytic transcripts is downstream of p53 stabilization in *Eftud2*-deficient cells and points to a pathway beyond the canonical p53 transcriptional response through which p53 can mediate cell-state changes in response to *Eftud2* deficiency.

To confirm that the changes in levels of glycolytic transcripts we observed are correlated with changes in cellular metabolism, we measured lactate levels in *Eftud2* KD mESCs in both p53-expressing and p53-null backgrounds. Lactate is an important cellular metabolite produced downstream of glycolysis, and we observed reduced lactate levels in *Eftud2* KD mESCs compared to nt-shRNA mESCs (Fig. S7A). This decrease was partially rescued in the p53-null background (Fig. S7A). Thus, decreased levels of select glycolytic transcripts point to changes in mESC metabolism in response to broad splicing perturbations.

Together, these *Eftud2* KD mESCs data revealed a signature of widespread metabolic downregulation at transcript level. We, therefore, hypothesized that *Eftud2* deficiency gives rise to a p53-stabilizing MDM2 isoform. In turn, this p53 stabilization might downregulate glycolytic transcripts. Interestingly, this signature of glycolytic transcript downregulation has not been observed in *Eftud2-*loss of function models (Beauchamp et al., 2021, 2022; Lei et al., 2017), underscoring the importance of studying disease-associated molecular signatures at varying degrees of splicing perturbation.

### Select glycolytic transcript downregulation is a p53-dependent common feature of splicing perturbations

We next hypothesized that the p53-dependent downregulation of glycolytic transcripts seen in response to *Eftud2* deficiency is a common response to broad splicing perturbations in mESCs. If our hypothesis is correct, we would expect to see a similar metabolic transcriptional signature in response to other broad splicing perturbations.

**Fig. 4.**
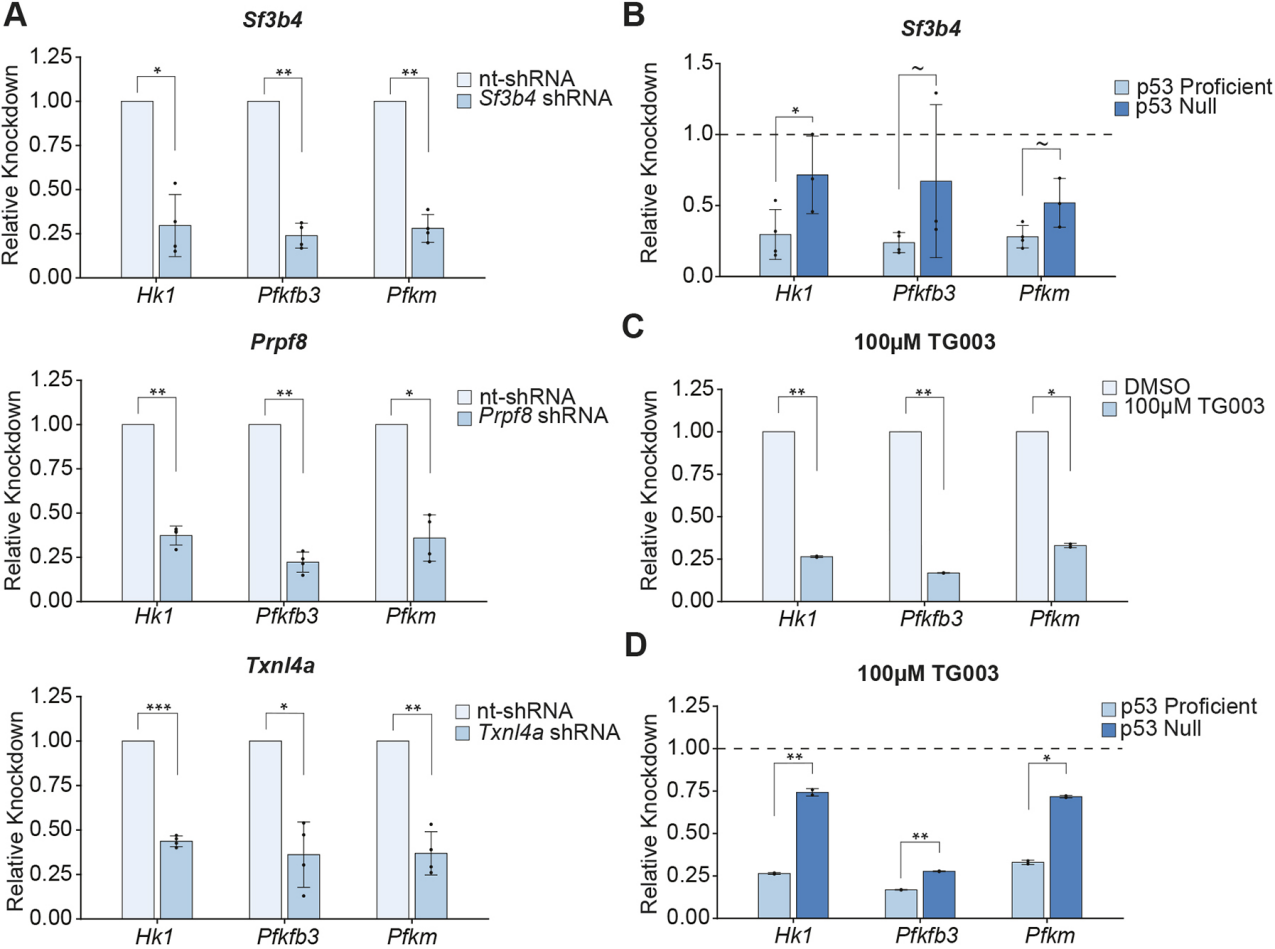
**Select glycolytic transcripts are downregulated under splicing perturbations.** (A) Quantification of qPCR of select glycolytic genes (*Hk1*, *Pfkfb3*, *Pfkm*) in mESCs infected with the stated shRNA. Bar graphs show 2^−ΔΔCt^, representing knockdown (KD) relative to control nt-shRNA treatment. Each biological replicate of shRNA KD was compaired pairwise to the corresponding nt-shRNA biological replicate to calculate KD levels, and each biological replicate is the average of three technical replicates. (B) Quantification of qPCR of select glycolytic genes in mESCs infected with *Sf3b4* shRNA, in p53-expressing (p53 Proficient) and p53-null mESC lines. Bar graphs show 2^−ΔΔCt^, representing KD relative to control nt-shRNA treatment in the given p53 status (denoted by dotted line). Each biological replicate of shRNA KD was compaired pairwise to the corresponding nt-shRNA biological replicate to calculate KD levels, and each biological replicate is the average of three technical replicates. (C) Quantification of qPCR of select glycolytic genes in mESCs treated with 100 µM TG003 for 3 days. Each biological replicate of TG003 treatment was compaired pairwise to the corresponding DMSO treatment biological replicate to calculate KD levels, and each biological replicate is the average of three technical replicates. (D) Quantification of qPCR of select glycolytic genes in mESCs treated with 100 µM TG003 for 3 days, in p53-expressing (p53 Proficient) and p53-null cell lines. Bar graphs show 2^−ΔΔCt^, representing KD relative to control DMSO treatment in the given p53 status (denoted by dotted line). Each biological replicate of TG003 treatment was compaired pairwise to the corresponding DMSO treatment biological replicate to calculate KD levels, and each biological replicate is the average of three technical replicates. Error bars represent ± standard deviation of biological replicates. Significance values determined by Student's *t*-test on paired ΔCt (A, C) or ΔΔCt (B, D) values. ΔCt values calculated by normalizing raw values to mitochondrial rRNA levels. *P<0.05, **P<0.01, ***P<0.001; ∼, not significant.

To examine whether downregulation of glycolytic transcripts is present in response to other splicing perturbations, we performed qPCR on select glycolytic transcripts after KD of the splicing factor genes *Txnl4a*, *Prpf8* or *Sf3b4* (Materials and Methods, see Infection with shRNAs). Specifically, we focused on three glycolytic transcripts – *Hk1*, which catalyzes the first step of glycolysis; *Pfkfb3*, which promotes allosteric activation of glycolytic flux and; *Pfkm*, one of the subunits that catalyzes a rate-limiting step in glycolysis (Lunt and Vander Heiden, 2011; Shi et al., 2017; Tanner et al., 2018). We found that KD of any of the above splicing factors always resulted in downregulation of glycolytic transcripts (Fig. 4A). To assay whether changes in the level of these transcripts correspond to changes in cellular metabolism, we measured lactate levels in mESCs expressing shRNAs targeting *Txnl4a*, *Prpf8* or *Sf3b4*. We found that all these splicing factor KDs reduce lactate levels compared to those of control nt-shRNA mESCs (Fig. S7B). In addition, we knocked down *Sf3b4* in p53-null mESCs and found that the degree of glycolytic transcript downregulation was reduced compared with that of p53-expressing cells (Fig. 4B). This p53-dependence is similar to our observation after *Eftud2* KD (Fig. 3F) and suggests that, in mESCs, p53-dependent downregulation of select glycolytic transcripts is a common response to splicing factor deficiency.

Furthermore, we found that treatment of mESCs with TG003 downregulates the level of glycolytic transcripts compared to that after control treatment, demonstrating that splicing stress in general leads to downregulation of select glycolytic transcripts in mESCs (Fig. 4C). Moreover, TG003 treatment of p53-null cells reduces the degree of glycolytic transcript downregulation (Fig. 4D). This finding emphasizes that downregulation of glycolytic transcripts was a response to splicing stress that is partially p53 dependent.

Together, our results revealed that downregulation of glycolytic transcript occurs downstream of p53 stabilization in the cellular stress response to broad splicing perturbations in p53-expressing mESCs.

### Inhibition of glycolysis phenocopies craniofacial defects of broad splicing perturbations in zebrafish embryos

The phenotypic consequences of broad splicing perturbations have been linked to p53-mediated apoptosis (Beauchamp et al., 2021, 2022; Lei et al., 2017; McElderry et al., 2019) but the link between splicing and other cell-state changes relevant to developmental disorders are unclear. Thus, we aimed to explore how the cellular response to broad splicing perturbations manifests itself by using a zebrafish developmental model.

**Fig. 5.**
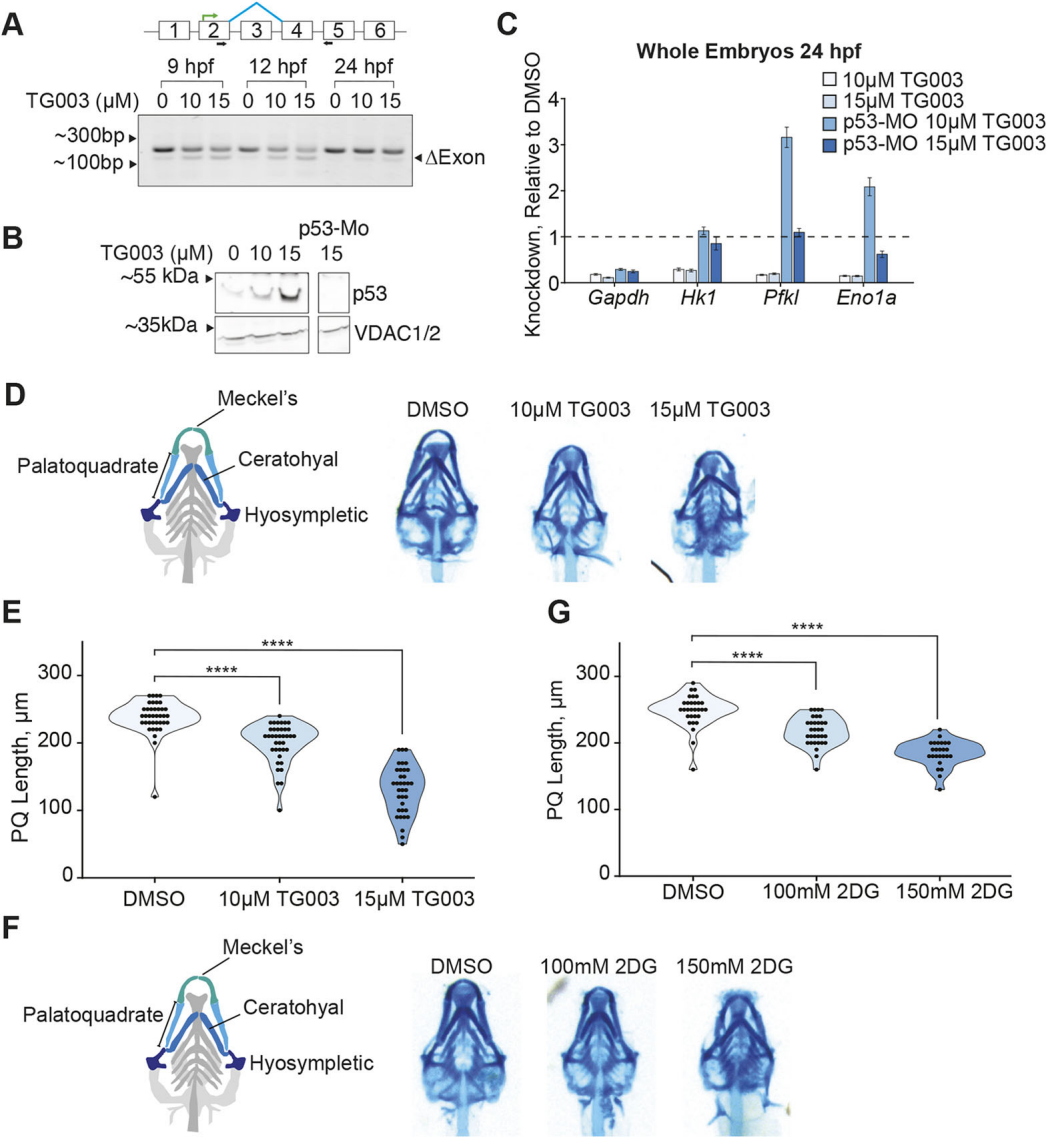
**Small-molecule glycolysis inhibition phenocopies small-molecule splicing inhibition in zebrafish.** (A) Top: Schematic of *mdm2* alternative splicing locus in zebrafish. Black arrows represent primer binding sites for sqRT-PCR. Green bent arrows represent start codons. Blue diagonal lines indicate exon-skipping event. Bottom: Agarose gel showing alternative splicing of *mdm2* in TG003-treated zebrafish. Semi-quantitative reverse transcription PCR of *mdm2* alternative splicing events in bulk zebrafish extracts (*n*=7 zebrafish/sample) using the primers designated by black arrows in the schematic. Zebrafish were treated at 6 hpf with DMSO, or with 10 or 15 µM of the splicing inhibitor TG003. Samples were collected 3, 6 and 18 h after treatement (i.e. 9, 12 and 24 hpf, respectively). (B) Western blot of p53 in bulk zebrafish protein extracts (*n*=8 zebrafish/sample) at 15 hpf treated with varying concentrations of TG003 as described in A, or p53-morphant zebrafish treated with 15 µM TG003. Levels of VDAC1/2 were used as loading control. (C) Quantification of qPCR for select highly expressed glycolytic transcripts in bulk zebrafish RNA extracts (*n*=7-8 zebrafish/sample) at 24 hpf, treated as described in A. Both p53-morpholino-injected and non-injected samples are shown. Bar graphs show 2^−ΔΔCt^, representing knockdown (KD) levels relative to control DMSO treatment in the given p53 status (denoted by the dotted line). ΔCt values normalized to mitochondrial RNA ND3-ND4L. Error bars represent the ±standard error of three technical replicates. (D) Representative images of Alcian Blue stained 5 dpf zebrafish that were treated with the indicated concentrations of TG003 at 6 hpf as described in A. (E,G) Quantification of palatoquadrate (PQ) bone length in zebrafish at 5 dpf, after treatment with TG003, 2DG or DMSO (control) as indicated. n∼30 per drug condition. ****P<0.0001. Significance values were determined by two-tailed Student's *t*-test (F) Schematic of the zebrafish viscerocranium (left) with first-arch structures indicated, and representative images of ventral views of the zebrafish viscerocranium (right) stained with Alcian Blue at 5 dpf after treatment with 2DG (100 or 150 mM) or DMSO as indicated at 6 hpf, showing underdeveloped lower jaw in 2DG-treated embryos and decreasing palatoquadrate length with increasing concentrations of 2DG.

To determine whether there is a cellular response to splicing perturbations in zebrafish, we treated zebrafish embryos 6 hours post fertilization (hpf) with varying concentrations of TG003 to broadly inhibit splicing. Consistent with our observations in mESCs, we found that an exon located at the 5′ end of *mdm2* is skipped in a concentration-dependent manner in response to treatment with TG003 (Fig. 5A). This *mdm2* alternative splicing event gave rise to a transcript that, as in mESCs, was predicted to produce an in-frame mdm2 isoform that lacks much of the sequence encoding the p53-binding domain. This finding emphasizes that *Mdm2* alternative splicing is a cellular response to broad splicing perturbations, which is conserved across species. Furthermore, we found that p53 is stabilized in zebrafish embryos treated with TG003, as commonly observed in zebrafish models of spliceosomopathies (Fig. 5B). We next investigated whether TG003-treated zebrafish exhibit the partially p53-dependent downregulation of select glycolytic transcripts. To this end, we injected 2-cell-stage zebrafish embryos with a previously used morpholino targeting p53 (Langheinrich et al., 2002), treated the embryos with TG003 and used whole embryos to perform qPCR for certain glycolytic transcripts. As in mESCs, this TG003 treatment resulted in the downregulation of many glycolytic transcripts in bulk zebrafish extracts (Fig. 5C). This downregulation was reduced in p53-morphant zebrafish that had been treated with TG003 (Fig. 5C), in which p53 stabilization was no longer observed (Fig. 5B), suggesting that, like in mammalian cells, metabolic cell-state changes occurred downstream of splicing perturbations and p53 stabilization in zebrafish. Altogether, these data demonstrated that, after treatment with TG003, a common response to broad splicing perturbations, as delineated in mESCs, also exists in zebrafish embryos.

As many spliceosomopathies present as defects in neural crest-derived cell types, we examined whether TG003-treated zebrafish embryos exhibited malformations in the neural crest-derived craniofacial cartilage. Indeed, as TG003 concentration increased, palatoquadrate length decreased, which is indicative of an underdeveloped lower jaw (Fig. 5D,E). This observed phenotype is not an overall scaling down of the craniofacial complex because the impact on palatoquadrate length was greater than the on other features of craniofacial morphology, such as jaw width (Fig. S8B). Furthermore, the palatoquadrate specificity of this phenotype was partially rescued in p53-morphant embryos, as a decrease in palatoquadrate length correlated more with changes of other craniofacial features (Fig. S8C). These findings are consistent with partial rescues observed in p53-deficient conditions for zebrafish models of other cranial malformations (Lau et al., 2016; Noack Watt et al., 2016), and support a role of p53 in cell-type-specific impacts of broad splicing perturbations. This craniofacial phenotype is analogous to the phenotype of an underdeveloped lower jaw seen in individuals with certain spliceosomopathies, as well as in animal models of these spliceosomopathies. It is noticeable that the phenotypes we observed are less severe than those seen in homozygous knock-out animal models, emphasizing the value of using small molecules to fine-tune the level of splicing disruption and produce more-moderate phenotypes that more resemble those seen in humans. In addition, by recapitulating the lower-jaw phenotype by using a small-molecule splicing inhibitor instead of targeting a single splicing factor, our data suggest that the observed phenotypes of spliceosomopathies are due to a common cellular response to splicing perturbations.

We next tested whether the defects we observed in the craniofacial cartilage of TG003-treated zebrafish embryos are correlated to impaired behavior of the neural crest during development. We did this by using hybridization chain reaction (HCR) to visualize the distribution of *sox10* mRNA in 15 hpf zebrafish embryos that had been treated with TG003 (15μM) or DMSO as control from 6 hpf. In addition to overall defects in embryo morphology, we observed a reduction of *sox10* signal in the anterior part of TG003-treated zebrafish as compared to control-treated embryos (Fig. S8D), suggesting that part of the craniofacial phenotype we observed is due to defects in early neural-crest development.

Finally, we hypothesized that the downregulation of glycolytic transcripts observed in response to treatment with TG003 may be partially responsible for the craniofacial phenotype observed in zebrafish, as cranial neural crest cells are uniquely dependent on aerobic glycolysis for successful EMT (Bhattacharya et al., 2020; Figueiredo et al., 2017). To further investigate this role of glycolysis in the craniofacial phenotype that arises under splicing perturbations, we treated 6 hpf zebrafish embryos with 2DG, a small molecule that inhibits early glycolysis at the hexokinase-catalyzed step (Aft et al., 2002). Notably, 2DG-treated embryos also exhibit a phenotype consistent with an underdeveloped lower jaw, similar to that observed in TG003-treated embryos, in which the palatoquadrate length decreased with increasing concentrations of 2DG (Fig. 5E,F). This phenotype does not correlate with broad changes within the remaining craniofacial structure (Fig. S8E), suggesting that palatoquadrate development was selectively impacted when glycolysis was inhibited. Thus, like splicing perturbations, inhibition of glycolysis in early zebrafish predominately impacted cell types derived from the neural crest.

Together, these results are consistent with a model in which at least part of the neural crest-cell-specific effects of splicing perturbations were mediated through downregulation of glycolytic transcripts and the resulting altered metabolic cell-state. More generally, our data obtained using zebrafish embryos support a model in which the common cellular response that arises upon broad splicing perturbations – consisting of *mdm2* alternative splicing, p53 stabilization and impaired metabolism – is responsible for some of the neural crest-cell-specific effects of spliceosomopathies.

### Glycolytic transcript downregulation is a common and persistent signature of long-term p53 stabilization in mESCs

Other craniofacial disorders similar to MFDM arise in response to other types of cellular perturbation, most notably following disruption of the ribosome-associated machinery, as seen in Treacher Collins syndrome (Dixon et al., 2006). The similarities between select ribosomopathies and spliceosomopathies are likely due to the uniquely heightened sensitivity of neural crest cells to p53 stabilization, as nucleolar stress also results in increased p53 stabilization (Olausson et al., 2012; Yang et al., 2018). Owing to these similarities, we hypothesized that downregulation of glycolytic transcripts is a common response to p53 stabilization in cells.

**Fig. 6.**
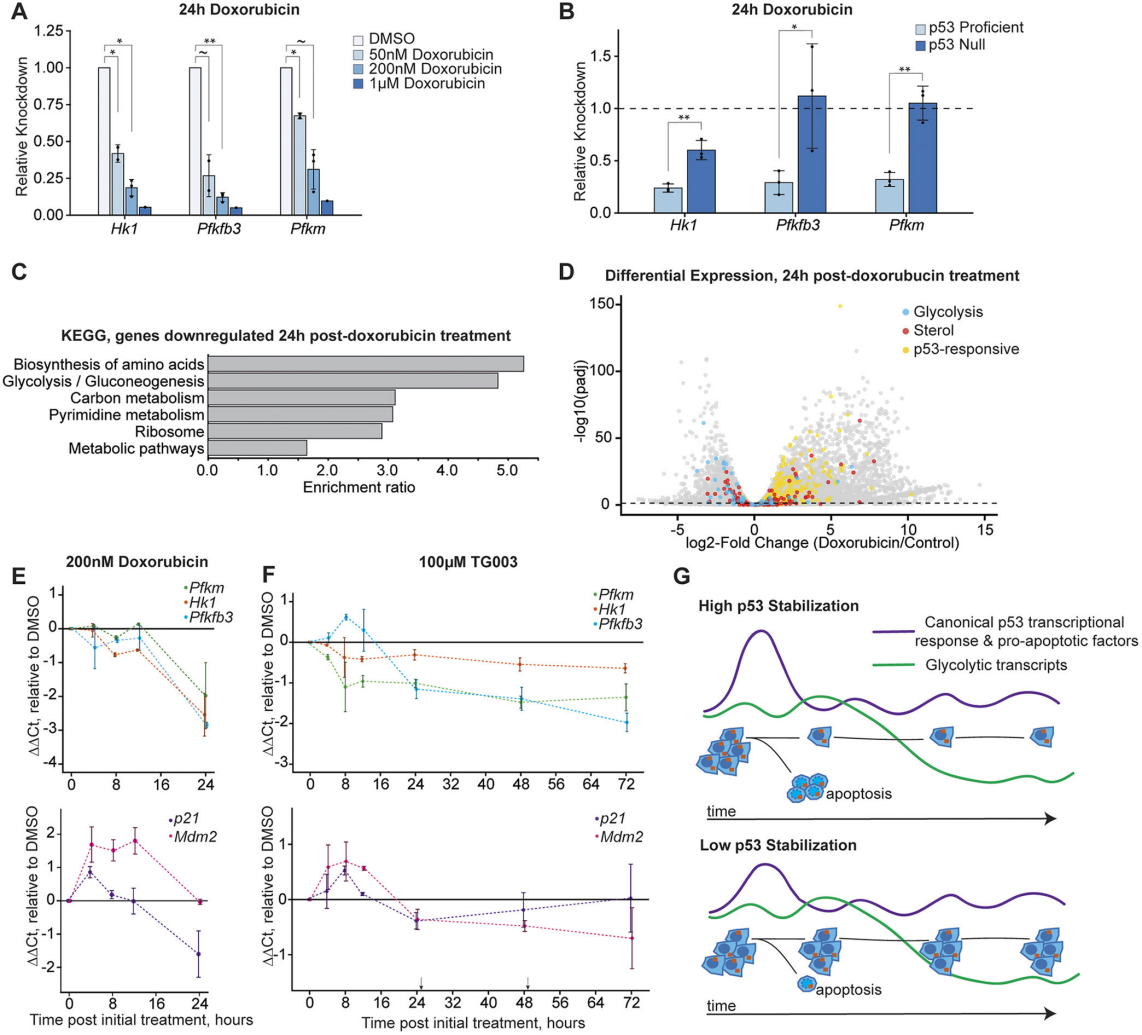
**Downregulation of select glycolytic transcripts is a common and prolonged response to p53 stabilization.** (A) Quantification of qPCR of select glycolytic genes (*Hk1*, *Pfkfb3*, *Pfkm*) in mESCs treated for 24 h with Doxorubicin at concentrations as indicated. Bar graphs show 2^−ΔΔCt^, representing knockdown (KD) relative to control DMSO treatment. Each biological replicate of Doxorubicin treatment was compared to DMSO treatment to calculate KD levels, and each biological replicate is the average of three technical replicates. Significance values determined by Student's *t*-test on ΔCt values. ΔCt values calculated by normalizing raw values to mitochondrial rRNA levels. *P<0.05; **P<0.01; ∼, not significant. (B) Quantification of qPCR of select glycolytic genes in mESCs treated with 200 nM Doxorubicin for 24 h in p53-expressing (p53 Proficient) and p53-null cells. Bar graphs show 2^−ΔΔCt^, representing KD relative to control DMSO treatment in the given p53 status (denoted by the dotted line). Each biological replicate of Doxorubicin treatment was compaired pairwise to the corresponding DMSO treatment biological replicate to calculate KD levels, and each biological replicate is the average of three technical replicates. Error bars represent ±standard deviation of biological replicates. Significance values determined by Student's *t*-test on ΔΔCt values. ΔCt values calculated by normalizing raw values to mitochondrial rRNA levels. *P<0.05, **P<0.01. (C) Over-representation analysis of Kyoto Encyclopedia of Genes and Genomes (KEGG) pathway genes downregulated in Doxorubicin-treated mESCs compared to those in control-treated mESCs as published previously (Grow et al., 2021). (D) Differential expression of all genes analyzed in C, comparing differential gene expression in Doxorubicin-treated mESCs and control-treated mESCs 24 h after treatment. Components of gene sets and their compilation are described under Compilation of gene set lists in Materials and Methods. Blue, genes involved in hexose sugar metabolism (Glycolysis); red, genes involved in sterol biosynthesis (Sterol); yellow, p53 transcriptional targets (p53 responsive). (E) Quantification of qPCR of the indicated genes of samples taken at the indicated timepoints. Cells were treated with 200 nM Doxorubicin at 0 h. ΔCt, ΔΔCt as defined in B. Values plotted are ΔΔCt, representing log2-Fold KD from DMSO. Plotted is the average of two biological replicates, error bars represent the ±standard deviation of biological replicates. Each biological replicate is the average of three technical replicates. (F) Quantification of qPCR of the indicated genes of samples taken at the indicated timepoints (*x*-axis). Cells were treated with 100 µM TG003 at 0 h, 24 h and 48 h (with samples of 24 h and 48 h timepoints taken before addition of TG003). ΔCt, ΔΔCt as defined in B. Values plotted are ΔΔCt, representing log2Fold KD from DMSO. Points are the average of two biological replicates. Error bars represent the ±standard deviation of biological replicates. Each biological replicate is the average of three technical replicates. (G) Model of cellular responses to p53 stabilization. Following high levels of p53 stabilization, most cells undergo p53-mediated apoptosis, leaving fewer remaining cells that undergo other p53-dependent cell-state changes. Following lower levels of p53 stabilization, fewer cells undergo apoptosis, with remaining cells showing lower levels of metabolic (glycolytic) transcripts.

To test this, we induced p53 stabilization with two small molecules, the MDM2 inhibitor Nutlin-3a or the anthracycline Doxorubicin (Vassilev et al., 2004; Aubel-Sadron et al., 1984). We then performed qPCR for select glycolytic transcripts, and found that, similar to what we had observed in response to splicing factor perturbations (Fig. 4), select glycolytic transcripts were downregulated after p53 stabilization (Fig. 6A, Fig. S9A). Furthermore, in p53-null cells, the downregulation of glycolytic transcripts following Doxorubicin treatment was reduced when compared to p53-expressing cells (Fig. 6B). In addition, we compared our data with previously published RNA-seq libraries constructed from Doxorubicin-treated mESCs (Grow et al., 2021). Analysis of these data showed a signature of glycolytic and metabolic transcript downregulation (Fig. 6C,D). This signature is no longer present in p53-null mESCs treated with Doxorubicin (Fig. S9B,C). These results show that downregulation of glycolytic transcripts is a common response to p53 stabilization in mESCs.

Although we observed parts of the p53 pathway being activated following *Eftud2* KD and Doxorubicin treatment (Figs 3B and 6D), we did not observe upregulation of apoptotic signaling pathways under these p53-stabilizing conditions, despite the well-known association with apoptosis (Beauchamp et al., 2021; Kurata et al., 2008; Lei et al., 2017). This is likely because both RNA-seq experiments were designed to capture long-term cell-state changes, and, thus, samples were not collected until at least 24 hours after treatment stabilizing p53. In response to many methods of p53 stabilization, the signatures of p53-mediated apoptosis and the canonical p53 transcriptional response would have waned by this timepoint (Jiménez et al., 2022; Purvis et al., 2012). These data suggest that downregulation of glycolytic transcripts is a slower, more persistent cellular response to p53 stabilization.

To further investigate the interplay between glycolytic transcript downregulation and canonical p53 target genes, we analyzed in more detail the timescale of transcript regulation in response to treatment with Nutlin-3a, Doxorubicin or TG003. Consistent with previous studies, the canonical p53 transcriptional targets of *Cdkn1a (p21)* and *Mdm2* were upregulated 4–8 h after drug treatment, and decreased quickly to pre-stabilization levels by 12 h (Fig. 6E,F, Fig. S9E). However, select glycolytic transcripts were not consistently downregulated until 24 h after drug treatment and this downregulation persisted well past the decline of p53-target genes (Fig. 6E,F, Fig. S9D,E).

Together, these data suggest a model whereby p53-mediated downregulation of glycolytic transcripts is a persistent cellular response to prolonged low-level p53 stabilization that arises under moderate splicing perturbations (Fig. 6G). Thereby, when splicing is broadly perturbed, p53 is stabilized and the canonical p53 transcriptional response is activated quickly, causing some cells to undergo apoptosis. The cells that do not undergo apoptosis enter a prolonged phase of reduced glycolytic transcript levels that can alter cell processes. The resulting cell-state changes could lead to the cell-type-specific phenotypes observed in disorders caused by widespread splicing disruption or in disorders caused by aberrant p53 stabilization, more generally.

## DISCUSSION

By examining splicing and transcriptomic alterations that occur in response to different disruptions of the splicing machinery, our work uncovered a unifying mechanism for a common cellular response to splicing perturbations. We defined a p53-stabilizing alternative splicing event in *Mdm2* as a common occurrence in response to broad splicing perturbations. The resulting stabilization of p53 propagates cell-state changes marked by transcript level downregulation of crucial genes involved in several metabolic pathways. The observed downregulation of transcript levels was not due to an alternative splicing event of a metabolic transcript, as previously observed when perturbing select splicing factors (Calabretta et al., 2016; Christofk et al., 2008; Jourdain et al., 2021; Su et al., 2017) but, rather, a widespread non-apoptotic response to p53 stabilization in cells when splicing in general was perturbed. These findings have important implications for how we view and understand broad splicing perturbations, and the pleiotropic effects of p53 in developmental disorders.

### Alternative splicing of Mdm2 serves as a sensor for the cellular response to broad splicing perturbations

The observed cellular responses to broad splicing perturbations exhibit many of the features ascribed to well-defined stress responses. For example, when ribosome biogenesis is disrupted, cells undergo nucleolar stress, a well-classified stress response that integrates the signals of stress at the p53-signaling node, and drives downstream cell-state changes mediated through p53 stabilization (Deisenroth and Zhang, 2010; Liao et al., 2021). Genetic models of nucleolar stress, termed ribosomopathies, exhibit partially p53-dependent phenotypes and, in some cases, primarily impact cell types derived from the neural crest, (Dixon et al., 2006; Falcon et al., 2022; Jones et al., 2008; Kampen et al., 2020), similar to what is observed in spliceosomopathies (Beauchamp et al., 2020). The parallels between the cellular and organismal responses to broad perturbations in the splicing machinery, and the better classified stress response of nucleolar stress suggest that splicing perturbations also result in a unified cellular stress response propagated through the p53 node. When classifying the cellular response to splicing perturbation, identification of the method through which the cell senses this stress and transmits the signal to p53 is of utmost importance.

Here, we found that a previously classified *Mdm2* splice variant commonly arises in mESCs and zebrafish embryos in response to broad splicing perturbations. Previous work has identified that this Mdm2 alternative splicing event arises after splicing perturbations of *dhx15* in zebrafish, *Eftud2* or *Snrpb* in mice and *SMN* in neuron models of spinal muscular atrophy (Alam et al., 2022; Beauchamp et al., 2021; McElderry et al., 2019; Van Alstyne et al., 2018). Our work discovered additional circumstances under which this event occurs to multiple splicing factors and broad small-molecule splicing inhibition in mESCs, and to broad small-molecule splicing inhibition in zebrafish. In addition, we found that the magnitude of the *Mdm2* alternative splicing event correlates with the degree of splicing perturbations. The Mdm2 isoform arising from this alternative splicing event lacks the p53-binding domain; thus, the increased frequency of this *Mdm2* alternative splicing event results in p53 stabilization (Cheng and Cohen, 2007; Giglio et al., 2010; Perry et al., 2000; Saucedo et al., 1999). From these data, we propose that under splicing stress, *Mdm2* acts as a sensor, transmitting splicing perturbations to p53 stabilization. This sensing mechanism, thereby, allows downstream cell state changes mediated by p53.

### Understanding cell-type-specific impact of splicing perturbations in developmental disorders

Although illuminating, previous literature investigating the impact of splicing factor deficiency on development made use of animal models that fail to recapitulate the haploinsufficiency seen in affected individuals with spliceosomopathies. As such, these animal models exhibit severe phenotypes and molecular signatures that are dominated by p53-mediated apoptosis (Beauchamp et al., 2021; Lei et al., 2017). However, it is likely that in response to milder levels of splicing factor perturbation these phenotypes are less severe and that other molecular signatures relevant to human disease exist besides apoptosis. In cells, we found that splicing perturbations result in downregulation of metabolic transcripts, one such molecular signature that could have relevance to disease phenotypes. Furthermore, by treating zebrafish embryos with varying concentrations of splicing inhibitor, we were able to observe milder organismal phenotypes and analyze what may occur in response to lower levels of splicing perturbation. Our data show that zebrafish treated with small-molecule splicing inhibitor phenocopy the craniofacial malformations observed in zebrafish treated with small-molecule glycolysis inhibitor. These data are consistent with previous findings, reporting that neural crest precursors of these craniofacial tissues are sensitive to reduced levels of glycolysis (Bhattacharya et al., 2020; Figueiredo et al., 2017), thus supporting a model whereby some of the phenotypes seen in conjunction with craniofacial spliceosomopathies are due to metabolic alterations.

Notably, although part the phenotype observed after treatment with small-molecule splicing inhibitors is p53-dependent, some aspects of the phenotypes due to splicing perturbations are p53 independent. There might be p53-independent metabolic alterations, such as alternative splicing of metabolic transcripts, which arise in response to broad splicing perturbations that impact neural crest cell behavior. Furthermore, it is possible that phenotypes arising from defects at different time points during development have different dependencies on p53 and cell-state changes propagated by p53. The use of a small-molecule splicing inhibitor, which allows the degree and timing of splicing perturbation to be modulated, will enable future studies to more closely investigate which cell types or at which point in time within the developing organism exhibit p53-independent or -dependent impacts of splicing perturbations.

Beyond the craniofacial structures derived from the neural crest, some splicing factor deficiencies result in retinitis pigmentosa, a disorder marked by defects and cell death in the photoreceptors of the retina (Arzalluz-Luque et al., 2021). Intriguingly, similar to the neural crest, photoreceptors have a heightened dependence on increased glycolysis to enable proper functioning, and disturbances of the delicate glycolytic balance of rods and cones result in photoreceptor death in retinitis pigmentosa (Petit et al., 2018). The downregulation of glycolytic transcripts we observed under splicing stress in this work could also reveal the mechanism through which some splicing perturbations cause retina-specific phenotypes.

Furthermore, previous work has found that, in cell culture, treatment of certain cancer types with splicing inhibitors in conjunction with traditional therapeutic measures increases the efficacy of cancer targeting (Bashari et al., 2023; Fan et al., 2011). Similar to the other cell types commonly targeted by splicing perturbations, cancer cells require increased glycolysis to undergo cell-state changes as they transform into aggressive cancer types. Thus, the downregulation of glycolytic transcripts we observed might explain why inducing splicing stress in cancer cells enhances treatment. It will be valuable to study additional ways of how to manipulate splicing stress in cancer cells to improve treatment options.

Beyond glycolytic transcript downregulation, we observed other metabolic transcript alterations when splicing was perturbed. Following *Eftud2* KD, we found that genes associated with sterol and cholesterol metabolism are broadly downregulated. Notably, defects in sterol biosynthesis have been linked to ten different syndromes, all of which display malformations of the craniofacial skeleton (Porter and Herman, 2011). Although the role of sterol-related pathways are less well understood than glycolysis in the context of cancer and development, it has been observed that disrupting cholesterol biosynthesis perturbs the Wnt signaling pathway during development, leading to craniofacial defects (Castro et al., 2020; Quintana et al., 2017). Altering sterol metabolism could be another mechanism through which cells regulate growth, differentiation and migration under splicing stress, and through which splicing perturbations have cell-type-specific impacts.

As we did not perform full metabolomics of cells undergoing splicing stress, our work is limited in understanding the scope of metabolic rewiring that occurs. Furthermore, the mechanism through which metabolic transcripts are downregulated in response to broad splicing perturbations is unclear. In human cell lines, p53 is known to downregulate glycolysis through the transcriptional activation of *TIGAR* and *MIR34A*, and transcriptional and post-transcriptional repression of various glycolytic genes (Bensaad et al., 2006; Kim et al., 2013; Liu et al., 2019). However, due to the widespread nature of the transcript downregulation we observed, it is likely that multiple mechanisms, both p53-dependent and -independent are at play to produce a concerted cell-state change during the splicing stress response. Further metabolic classification is merited to study the altered energetic state, the mechanisms through which these changes arise and how p53 impacts these changes.

### Re-examination of the cellular response to p53 stabilization

p53 serves as the central node in many cellular stress responses besides splicing stress, and propagates widespread cellular changes downstream the initial stress trigger (Kaiser and Attardi, 2018). Although the method through which p53 is stabilized is unique to splicing stress, we also detected a similar downstream signature of glycolytic transcript downregulation in response to other inducers of p53 stabilization. Thus, our work raises the possibility that phenotypes resulting from aberrant p53 stabilization during other stress responses are attributed to metabolic cell-state changes.

Although p53 produces pleiotropic effects in cells, some cell types, including those that arise from the neural crest, exhibit unique sensitivities to a subset of the responses downstream of p53 (Bowen et al., 2021; Calo et al., 2018). By using mESCs, we captured a chronic non-apoptotic cell-state change that occurred after prolonged p53 stabilization, and which might be relevant to disorders of p53 stabilization during which cells impacted are less sensitive to p53-mediated apoptosis but more sensitive to metabolic perturbations. For instance, disruption of the apoptotic p53 response partially rescues phenotypes in the neural craniofacial lineages but does not rescue phenotypes in the chondrocyte lineages, even though p53 is aberrantly stabilized in all lineages (Bowen et al., 2021). Our data illuminate the possibility that the p53-mediated phenotypes in chondrocytes and other cell types sensitive to levels of metabolites are due to metabolic alterations, rather than apoptosis. Our work is not in conflict with other models of p53 stabilization in disease and development but, by considering multiple facets of the cellular response to p53 stabilization, we are afforded a more unifying view that could help explain complex organismal phenotypes.

Together, our work demonstrates a yet uncovered pathway through which cells respond to splicing stress and, more generally, prolonged periods of sub-lethal levels of aberrant p53 stabilization. Our work might pave the way for further studies examining the cell-type-specific nuances to the splicing stress response, and the interplay between the p53 pathway and metabolic alterations. Furthermore, our work underscores the importance of considering cell type, magnitude and duration of p53 stabilization when studying perturbed cell states in disease.

## MATERIALS AND METHODS

### Cell culture

R1 mouse embryonic stem cells (mESCs, American Type Culture Collection (ATCC), #SCRC-1011) were cultured in 5% CO_2_ on six-well plates (Genesee Scientific, #25-105), coated with poly-L-ornithine (Millipore-Sigma, P3655) and laminin (Corning/Fisher, #CB-40232) or 10 µg/ml fibronectin (Fisher Scientific, FC01010MG), in N2B27 medium supplemented with 1 µM PD0325901 (Sigma-Aldrich, #PZ0162), 3.3 µM CHIR99021 (Sigma-Aldrich, #SML1046), and 1% leukemia inhibitory factor (LIF; generated in-house from COS-1 cells engineered to secrete LIF) as previously described (Thomson et al., 2011; Ying et al., 2008; Ying and Smith, 2003).

N2B27 medium was composed of a 1:1 mixture of Dulbecco's modified Eagle medium (DMEM)/F12 (Thermos Fisher Scientific, #11320033) and Neurobasal medium (Thermo Fisher Scientific, #21103049), 1:100 N2 NeuroPlex medium (Gemini Bio, #400-163), 1:50 B27 without vitamin A (Gemini Bio, #400-161), 1:100 MEM non-essential amino acids solution (Thermo Fisher Scientific, #11-140-050), 1% penicillin/streptomycin/glycine (PSG, Gibco, #10378-016), 0.5% GlutaMAX supplement (Thermo Fisher Scientific, #35050061), 0.5% sodium pyruvate (Thermo Fisher Scientific, #11360070), 0.1% 2-mercaptoethanol (Gibco, #21985-023), and 0.1% w/v Albumax II (Thermo Fisher Scientific, #11021029).

HEK293FT cells (Thermo Fisher Scientific, #R70007) were cultured on tissue-culture plates (Genesee Scientific, #25-105) in DMEM (Genesee Scientific, #25-500)+10% fetal bovine serum (FBS) (Gemini Bio-products, 100-106)+1% PSG.

All cell lines were passaged with trypsin (Gibco, #25200072). All cell lines used were mycoplasma free.

mESCs were treated with indicated concentrations of TG003 (Selleck Chemical, #S7320), Doxorubicin (Cell Signaling Technology, #5927) or Nutlin-3a (Selleck Chemical #675576-98-4).

### Generation of p53-null cell lines

sgRNAs that target the CRISPR machinery to an early exon of p53 (Table S1) were ligated into pX459 (Addgene #62988). Plasmids were transfected into mESCs with Lipofectamine 2000 (Thermo Fisher Scientific, #11668027) and Opti-MEM (Thermo Fisher Scientific, #31985070). mESCs were selected on 1µg/mL puromycin (Gibco, #A11138-03) for 1 day, colonies were picked, genomic DNA screened for frameshift mutations predicted to cause premature termination codons and NMD-targeted p53 transcripts. p53-null status was validated using western blotting.

### RNA extraction, cDNA synthesis

RNA was extracted using Trizol (Thermo Fisher Scientific, #15-596-018), chloroform and subsequent isopropanol precipitation. cDNA was synthesized using Superscript Vilo MasterMix (Invitrogen #11755050).

### qPCR and sqRT-PCR

qPCR primers are listed in Table S1. qPCR was performed on Lightcycler machines, with KAPA SYBR Fast qPCR mastermix (Roche, #KK4600). Raw partial cycle threshold (ΔCt) values and standard errors of biological replicates are listed in Table S2. To calculate KD levels relative to control samples, 2^−∆∆Ct^ was calculated, where ∆∆Ct is the difference in ∆Ct values between control and experimental samples. Significance was determined by two-sided Student's *t*-test on ∆Ct or ∆∆Ct values, depending on the comparison being made. sqRT-PCR was performed with primers listed in Table S1, band intensities were quantified in Fiji by taking the sum of the pixel intensities within the band region using the gel analyzer tool.

### Lactate measurement in cells

Cells were lysed on ice and lactate production was measured using the L-Lactate Assay Kit (Colorimetric/Fluorometric) (Abcam, #ab65330) according to manufacturer instructions. All lactate measurements were standardized to protein concentration in the lysate, as measured with Pierce Detergent Compatible Bradford Assay Kit (Thermo Fisher Scientific, #23246).

### Western blotting

Cells were scraped into cold PBS, and resuspended in RIPA buffer supplemented with PMSF (Thermo Fisher Scientific, #36978) and cOmplete protease inhibitor cocktail (Sigma Aldrich, #11697498001). Extracts were agitated in cold, centrifuged, and supernatants used for western blotting. Blots were run in T-G running buffer (1× Tris-Glycine+1% SDS), transferred into 1× T-G+20% methanol, and blocked in 5% milk in PBS-Tween. Membranes were incubated overnight at 4°C with primary antibody, washed with PBS-Tween, and incubated for 1 h at room temperature with secondary antibody. Membranes were washed, secondary antibody was detected by chemiluminescence using SuperSignal West Femto maximum sensitivity substrate (Thermo Fisher Scientific, #34096), and imaged with Azure Biosystems C Series Capture.

Antibodies used were: anti-p53 (1:1000, Cell Signaling #2524 1C12), anti-β-tubulin (1:2000, Thermo Fisher Scientific, #ma5-15308), anti-EFTUD2 (1:2000, Abcam, #ab72456), zebrafish anti-p53 (1:500, GeneTex, #gtx128135), anti-VDAC1/2 (1:500, Cell Signaling, #10866-1-AP), goat anti-rabbit HRP (1:10,000, Invitrogen, #32260), goat anti-mouse HRP (1:10,000, Invitrogen, #32230).

### Infection with shRNAs

shRNAs targeting *Eftud2* (shRNA1 – Sigma Aldrich, #TRCN0000294567, shRNA2 – Sigma Aldrich, #TRCN0000306704), *Sf3b4* (Sigma Aldrich, #TRCN0000379192), *Txnl4a* (Sigma Aldrich, #TRCN0000123687), *Prpf8* (Sigma Aldrich, #TRCN0000109106) or non-targeting (nt-) shRNA (Addgene, 30323) were transfected with lentiviral envelope and packaging plasmids (Addgene, psPAX2 12260, pMD2.G 12259) into HEK293FT cells using Lipofectamine 2000 (Thermo Fisher Scientific, #11668027). Virus-containing medium from HEK293FT cells was collected and used to infect mESCs on two subsequent days. Each day mESCs were incubated in a mix of virus-containing and standard mESC medium (1:1) for 4 hours before changing the medium to standard mESC medium alone. Cells were then washed with cold PBS and collected for analyses.

### RNA sequencing and analysis

cDNA libraries were prepared using NEBNext Ultra II with Poly(A) Selection (New England Biolabs, #E7760S). RNA-Seq was performed using Illumina NextSeq500, 100 bp paired-ends reads. Quality Control was performed using FastQC.

Sequences were aligned to the reference genome assembly mouse mm10 genome (GRCm38) by using STAR Aligner (Dobin et al., 2013), using alignEndsType/EndToEnd to disable soft-clipping for downstream splicing analyses. Aligned reads were sorted with featureCounts (Liao et al., 2014), specifying for paired end reads.

Differential expression analysis was done via DESeq2 (Love et al., 2014; https://bioconductor.org/packages/release/bioc/html/DESeq2.html), excluding low-count transcripts. The DESeq2 matrix is presented in Table S3. Alternative splicing was assayed by using rMATS (Shen et al., 2014; https://rnaseq-mats.sourceforge.io/), with significance cut-offs for analyses being a false discovery rate (FDR)<0.01. Filtered rMATS output is presented in Table S4.

### Gene Ontology and pathway analysis

Gene Ontology (GO) over-representation analysis and PANTHER Pathway analysis were completed using WebGestalt (Liao et al., 2019).

Background reference sets were compiled from all genes detected in DESeq2 (https://bioconductor.org/packages/release/bioc/html/DESeq2.html), all genes detected in rMATS output and all genes detected in rMATS Exon Skipping output.

For differential expression analysis, the top 1000 most significantly down- or upregulated genes were plotted. For the overlap sets, all down or all up-regulated genes were used as input. For rMATS analysis, genes that had at least one significant Alternative splicing or skipped exon event were included. We did not account for multiple alternative splicing events in the same gene.

Analysis was performed using the Bonferroni multiple test correction, with an FDR cut-off of 0.05. Terms with FDR<0.05 were sorted on enrichment ratio, and the top five to ten terms are displayed. Gene Ontology and PANTHER categories were removed from analyses if a given category contained <5 or >2000 genes.

### Compilation of gene set lists

The glycolysis GO terms GO:0030388, GO:0006003, GO:0006002, GO:0019255, GO:0051156, GO:0019682, GO:0060096 were combined to encapsulate all genes in pathway. We excluded terms from the resulting list whose designated role was only regulatory or only as ‘Pentose Phosphate Pathway’. This yielded a list of 64 genes we designated to be core factors in the glycolysis pathway.

The sterol gene list was compiled as a combination of GO:0016125, GO:0008203, GO:0016126, GO:0006695, excluding terms whose role was only designated as regulatory.

The p53 responsive gene list was compiled from previous literature, specifically focusing on genes known to be positively regulated by and promoters bound by p53 in mESCs (Bowen et al., 2019; Lee et al., 2010; Li et al., 2012).

### Zebrafish handling and husbandry

Zebrafish were handled according to the vertebrate animal handling protocol. AB (wild-type) zebrafish were used for all experiments. For morpholino experiments, 2-cell-stage zebrafish were injected with 1–2 nl 500 µM p53-morpholino (5′-GCGCCATTGCTTTGCAAGAATTG-3′, GeneTools PCO-ZebrafishP53-100). Non-injected embryos were used as control.

At 6 hpf, zebrafish were treated with TG003 or 2-deoxy-D-glucose (2DG, Sigma-Aldrich, # D8375-1G). Water quality conditions were: dissolved oxygen = 6.50±0.25 mg/L, salinity = 0.0 practical salinity unit (PSU) pH 7.2, conductivity = 520µS/cm), temperature = 28°C. Drug was washed out at 24 hpf (i.e.18 hours after treatment) and no additional drug was added. Water was changed every subsequent day, and fish were collected at 5 days post fertilization (dpf) for cartilage staining.

Fish were fixed, bleached and stained with Alcian Blue as described previously (Dingerkus and Uhler, 1977; Sakata-Haga et al., 2018). Fish were imaged using a Leica M205 FCA stereo microscope, using the same settings for all samples (bright-field, exposure = 1 ms). Lengths of craniofacial structures were analyzed using Fiji, by an investigator to whom the treatment groups had been anonymized.

### Hybridization chain reaction

15 hpf zebrafish embryos were fixed overnight in 4% paraformaldehyde at 4°C and dehydrated in methanol. Embryos were rehydrated and hybridization chain reaction (HCR) was carried out per manufacturer instructions (Molecular Instruments) with the *sox10* (NM_131875) probe. Samples were imaged on an Olympus FV1200 confocal laser scanning microscope.

### Statistics

Statistics for RNA-Seq analysis were performed using DESeq2 and rMATS, with *P*-value cut-offs set at padj<0.05 and FDR<0.01, respectively. All other statistical analyses were carried out in R. For qPCR analysis, significance was assayed using two-tailed Student's *t*-test on biological replicates (each biological replicate is the average of three technical replicates) with *P*<0.05 deemed significant. For analysis of zebrafish craniofacial structure measurements, significance was assayed using two-tailed Student's *t*-test with *P*<0 .05 deemed significant.

## Supplementary Material

10.1242/dmm.050356_sup1 Supplementary information

Table S3. DESeq2 output

Table S4. rMATS filtered output
