# Supplementary material for: A common cellular response to broad splicing perturbations is characterized by metabolic transcript downregulation driven by the Mdm2–p53 axis
Source: Dis Model Mech. 2024 Mar 1;17(2):dmm050356. doi: 10.1242/dmm.050356 (PMC10924232; doi:10.1242/dmm.050356)
Supplement: Supplementary information [file dmm-17-050356-s1.pdf]

Materials & Methods

Cell Culture

R1 Mouse embryonic stem cells (mESCs, ATCC SCRC-1011) were cultured in 5% CO2 on 6-well plates (Genesse Scientific, 25-105), coated with Poly-L-Ornithine (Millipore-Sigma, P3655) and Laminin (Corning/Fisher, CB-40232), or 10µg/mL Fibronectin (Fisher Scientific, FC01010MG), in N2B27 media supplemented with 1µM PD0325901 (Sigma-Aldrich, PZ0162), 3.3µM CHIR99021 (Sigma-Aldrich, SML1046), and 1% Lif (Generated in-house from COS-1 cells engineered to secrete Lif) as previously described (Thomson et al., 2011; Ying et al., 2008; Ying and Smith, 2003)

N2B27 media is composed of a 1:1 mixture of DMEM/F12 (Thermo Fisher 11320033) and Neurobasal (Thermo Fisher 21103049), 1:100 N2 Neuroplex (Gemini Bio, 400-163), 1:50 B27 without Vitamin A (Gemini Bio, 400-161), 1:100 MEM NEAA (Thermo Fisher, 11-140-050) , 1% Penicillin/Streptomycin/Glycine (PSG, Gibco, 10378-016), .5% Glutamax Supplement (Thermo Fisher, 35050061), .5% Sodium Pyruvate (Thermo Fisher 11360070), .1% 2-Mercaptoethanol (Gibco, 21985-023), and .1% w/v Albumax II (Thermo Fisher 11021029).

HEK293FT cells (ATCC) were cultured on TC-coated plates in Dulbecco's Modified Eagle Medium (DMEM, Genessee Scientific, 25-500) + 10% Fetal bovine serum (FBS, Gemini Bio-products, 100-106) + 1% PSG.

All cell lines were passaged with trypsin (Gibco, 25200072). All cell lines used were mycoplasma-free.

mESCs were treated with indicated concentrations of TG003 (Selleck Chemical, S7320), Doxorubicin (Cell Signaling Technology, #5927), or Nutlin-3a (Selleck Chemical 675576-98-4).

p53-null cell lines generation

sgRNAs that target the CRISPR machinery to an early exon of p53 (Table S1) were ligated into pX459 (Addgene #62988). Plasmids were transfected into mESCs with Lipofectamine 2000 (Thermo Fisher, 11668027) and Opti-MEM (Thermo Fisher, 31985070). mESCs were selected on 1µg/mL puromycin (Gibco, A11138-03) for 1 day, colonies were picked, genomic DNA screened for frameshift mutations predicted to cause premature termination codons and NMD-targeted p53 transcripts , and p53 null status validated via Western Blot.

RNA Extraction, cDNA Synthesis

RNA was extracted using Trizol (Thermo Fisher 15-596-018), chloroform and subsequent isopropanol precipitation. cDNA was synthesized using Superscript Vilo MasterMix (Invitrogen 11755050).

qPCR and sqRT-PCR

qPCR primers are listed in Supplementary Table S1. qPCR was performed on Lightcycler machines, with KAPA SYBR Fast qPCR mastermix (Roche, KK4600). Raw ∂Ct values and standard errors for

Disease Models & Mechanisms • Supplementary information

biological replicates listed in Supplementary Table S2. sqRT-PCR was performed with primers listed in Table S1, band intensities quantified in Fiji by taking the sum of the pixel intensities in the band region using the gel analyzer tool.

Lactate Measurements in Cells

Cells were lysed on ice and lactate production was measured using L-Lactate Assay Kit (Colorimetric/Fluorometric) (Abcam, ab65330) according to manufacturer instructions. All lactate measurements are standardized to protein concentration in the lysate, as measured with Pierce Bradford Reagent (ThermoFisher Sceintific 23246).

Western Blotting

Cells were scraped in cold PBS, and resuspended in RIPA buffer supplemented with PMSF (ThermoFisher Scientific, 36978) and cOmplete protease inhibitor cocktail (Sigma Aldrich, 11697498001). Extracts were agitated in cold, centrifuged, and supernatants used for Westerns. Blots were run in T-G running buffer (1x Tris-Glycine + 1% SDS), transferred in 1x T-G + 20% Methanol, and blocked in 5% milk in PBS-Tween. Membranes were incubated overnight at 4ºC in primary antibody, washed with PBS-Tween, and incubated for 1 hour at room temperature in secondary antibody. Membranes were washed, secondary antibody detected via Chemi-luminescent detection with SuperSignal West Femto (ThermoFisher Scientific 34096),and imaged with Azure Biosystems C Series Capture.

Antibodies used - p53 (1:1000,Cell Signaling #2524 1C12), β-TUBULIN (1:2000, ThermoFisher Scientific ma5-15308), EFTUD2 (1:2000, Abcam ab72456), Zebrafish p53 (1:500, Genetex gtx128135), VDAC1/2 (1:500, Cell Signaling 10866-1-AP), Goat anti-Rabbit HRP (1:10000, Invitrogen 32260), Goat anti-mouse HRP (1:10000, Invitrogen 32230).

Infection with shRNAs

shRNAs targeting *Eftud2* (shRNA1 – Sigma Aldrich TRCN0000294567, shRNA2 – Sigma Aldrich TRCN0000306704), *Sf3b4* (Sigma Aldrich, TRCN0000379192), *Txnl4a* (Sigma Aldrich, TRCN0000123687), and *Prpf8* (Sigma Aldrich, TRCN0000109106) and Non-targeting shRNA (Addgene, 30323) were transfected, along with lentiviral envelope and packaging plasmids (Addgene, psPAX2 12260, pMD2.G 12259) into HEK293FTs with Lipofectamine 2000 (Thermo Fisher, 11668027). Viral-containing media from HEK293FTs was collected, and incubated on mESCs for 4 hours, on 2 consecutive days. mESCs were selected under 2ng/µL of puromycin for 3 days. Cells were washed with cold PBS, and collected for downstream analyses.

RNA-Sequencing and Analysis

cDNA libraries were prepared using NEBNext Ultra II with Poly(A) Selection (New England Biolabs, E7760S). RNA-Seq was performed using Illumina NextSeq500, 100bp paired-ends reads. Quality Control was performed using FastQC.

Sequences were aligned to the mm10 genome using STAR Aligner (Dobin et al., 2013), using alignEndsType EndToEnd to disable soft-clipping for downstream splicing analyses. Aligned reads were sorted with featureCounts (Liao et al., 2014), specifying for paired end reads.

Differential expression analysis was done via DESeq2 (Love et al., 2014), excluding low-count transcripts. The DESeq2 matrix is presented in Supplementary Table S3. Alternative splicing was

Disease Models & Mechanisms • Supplementary information

assayed using rMATS (Shen et al., 2014), with significance cutoffs for analyses FDR < 0.01. Filtered rMATS output is presented in Supplementary Table S4.

Gene Ontology & Pathway Analysis

Gene Ontology Over-Representation Analysis and PANTHER Pathway analysis were completed using WebGestalt (Liao et al., 2019).

Background reference sets were compiled from all genes detected in DESeq2, all genes detected in rMATS output, or all genes detected in rMATS Exon Skipping output.

For Differential Expression analysis, the top 1000 most significantly down or up-regulated genes were plotted. For the overlap sets, all down or all up-regulated genes were used as input. For rMATS analysis, genes that had at least one significant Alternative splicing or SE event were included. We did not account for multiple AS events in the same gene.

Analysis was performed using the Bonferroni multiple test correction, with an FDR cutoff of 0.05. Terms with FDR < 0.05 were sorted on enrichment ratio, and the top 5-10 terms are displayed. Basic settings were set at Min # genes/cat 5; max 2000.

Compilation of gene set lists

Glycolysis GO terms GO:0030388, GO:0006003, GO:0006002, GO:0019255, GO:0051156, GO:0019682, GO:0060096 were combined to encapsulate all genes in pathway. We excluded terms from the resulting list whose role was only designated as regulatory or only designated as "Pentose Phosphate Pathway". This yielded a list of 64 genes we designated as core factors in the glycolysis pathway.

Sterol gene list was compiled as a combination of GO:0016125, GO:0008203, GO:0016126, GO:0006695 excluding terms whose role was only designated as regulatory

p53 responsive gene list was compiled from previous literature, specifically focusing on genes known to be positively regulated and promoters bound by p53 in mESCs (Bowen et al., 2019; Lee et al., 2010; Li et al., 2012)

Zebrafish handling & husbandry

Zebrafish were handled according to the vertebrate animal handling protocol. AB (wild-type) zebrafish were used for all experiments. For morpholino experiments, 2-cell-stage zebrafish were injected with 1-2nl 500µM p53-morpholino (GCGCCATTGCTTTGCAAGAATTG, GeneTools PCO-ZebrafishP53-100). Uninjected embryos were used as controls.

At 6 hpf, zebrafish were treated with TG003 or 2-Deoxy-D-Glucose (2DG, Sigma-Aldrich D8375-1G) in fish facility water. Drug was washed out at 24 hpf (18 hours-post-treatment), and no additional drug was added. Water was changed every subsequent day, and fish were collected at 5 dpf for cartilage staining.

Fish were fixed, bleached, and stained with Alcian Blue as described previously (Dingerkus and Uhler, 1977; Sakata-Haga et al., 2018). Fish were imaged using a Leica M205 FCA stereo microscope, with the same settings for all samples. Craniofacial structure length was analyzed, blind to treatment groups, on Fiji.

Hybridization Chain Reaction

15 hpf zebrafish embryos were fixed overnight in 4% PFA at 4ºC, and dehydrated in methanol. Embryos were rehydrated, and HCR was carried out per manufacturer instructions (Molecular Instruments) with the *sox10* (NM_131875) probe. Samples were imaged on an Olympus FV1200 Laser Scanning Confocal Microscope.

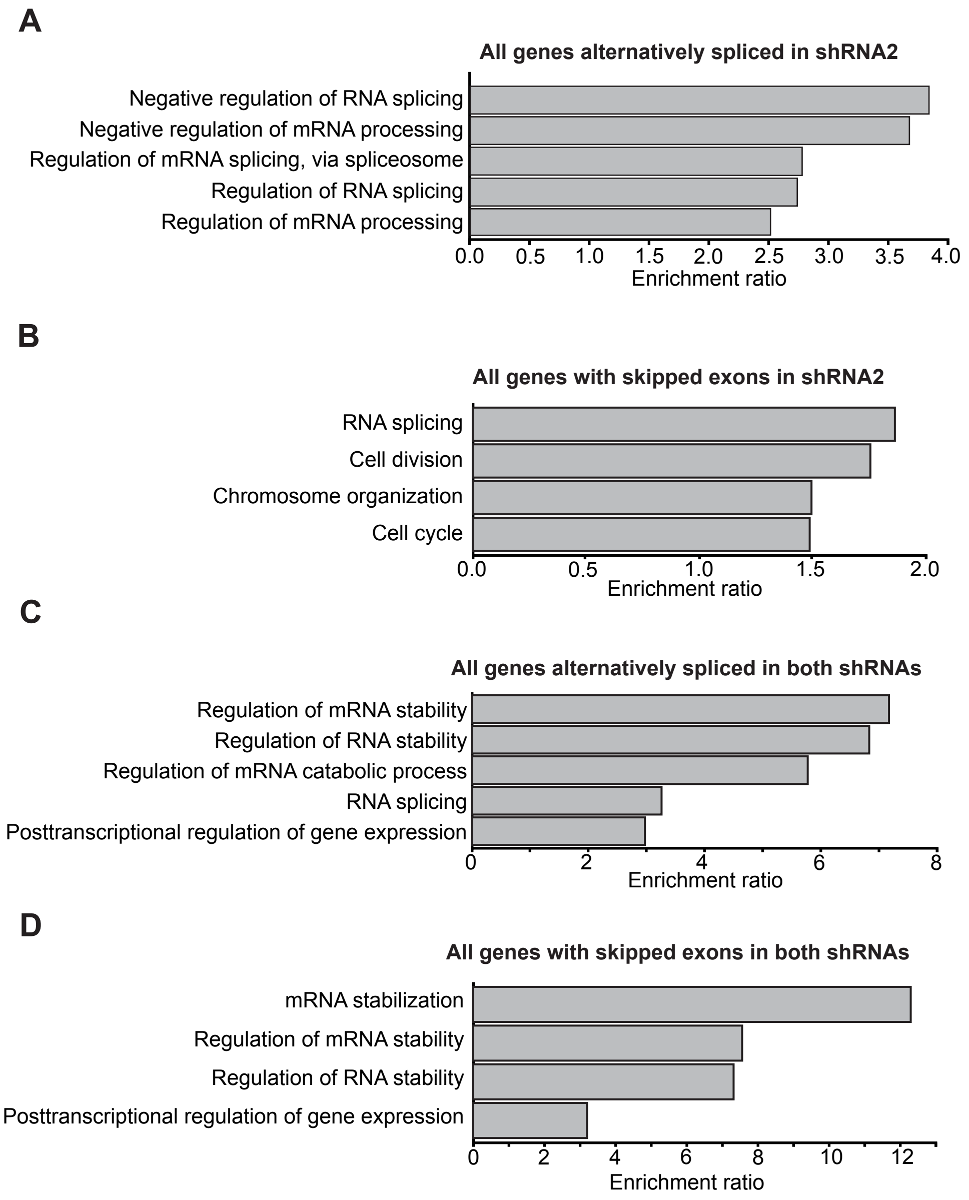

Fig. S1. Gene Ontology analysis of alternatively spliced genes under *Eftud2* KD

A. Gene Ontology Biological Process of the set of genes alternatively spliced (all five types of alternative splicing detected in rMATS) under shRNA2 KD. There are no significant GO terms for shRNA1 KD.
B. Gene Ontology Biological Process of the set of genes containing a significant Exon-skipping event under shRNA2 KD. There are no significant GO terms for shRNA1 KD.
C. Gene Ontology Biological Process of the set of genes alternatively spliced (all five types of alternative splicing detected in rMATS) under both shRNAs used when compared to control.
D. Gene Ontology Biological Process of the set of genes containing a significant Exon-skipping event under both shRNAs used when compared to control.

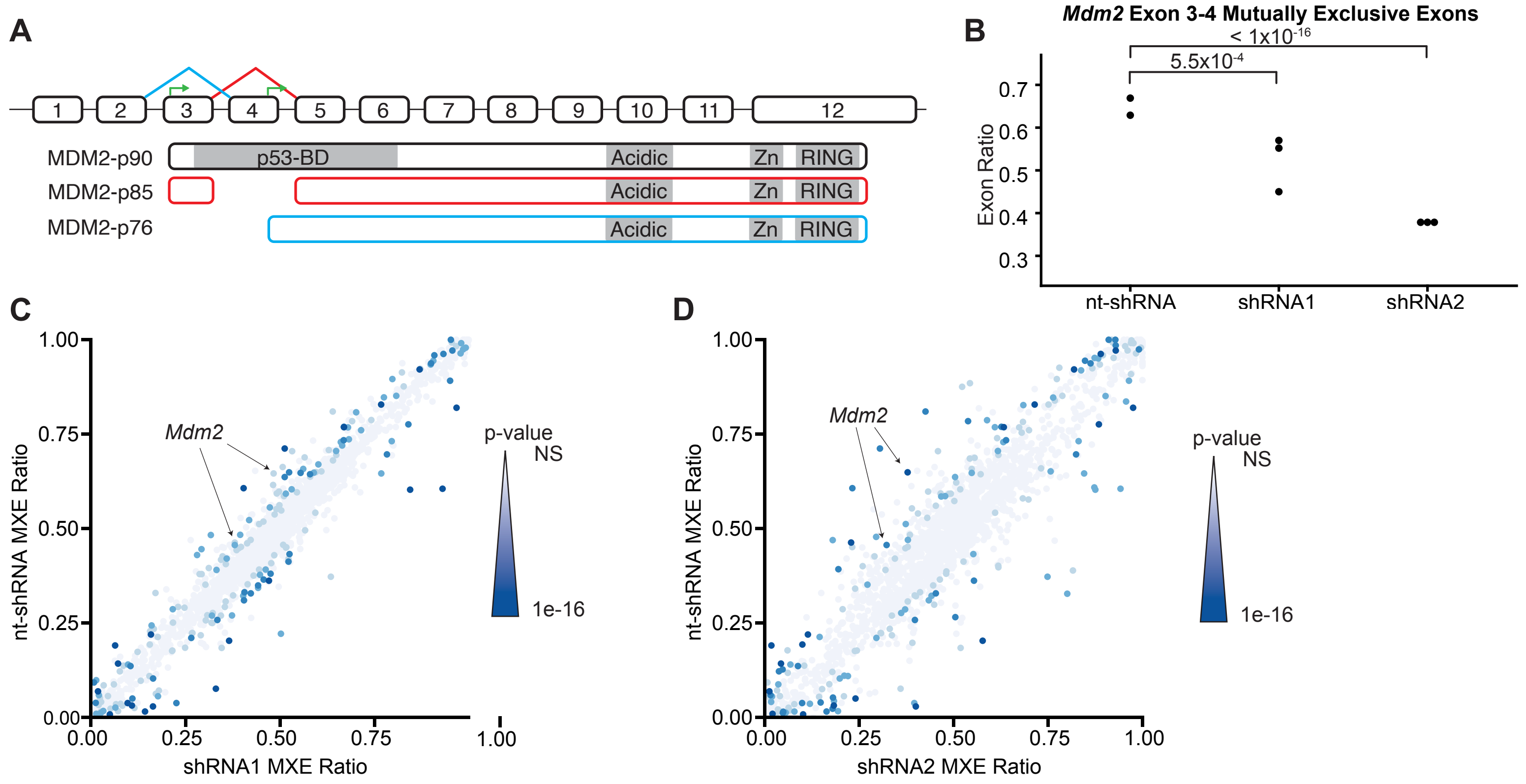

Fig. S2. *Mdm2* Mutually Exclusive Exon event detected under *Eftud2* KD

A. Diagram of the mutually exclusive exon event (MXE) detected by rMATs in *Mdm2* and resulting isoforms with select protein domains annotated. The MXE event gives rise to isoforms that lack portions of the p53-Binding Domain. p53-BD, p53-Binding Domain; Acidic, Acidic Domain; Zn, Zinc Finger; RING, RING Domain.
B. The ratio of Exon 3 inclusion and Exon 4 skipping to Exon 3 skipping and Exon 4 inclusion as detected by rMATs.
C. "Inclusion" values of MXE events as detected by rMATS in shRNA1 (x-axis) and nt-shRNA (y-axis). Darker colors indicate more significant p-values. Both *Mdm2* MXE events indicated are similar events, defined as separate events by rMATS due to slight differences in the upstream exon.
D. "Inclusion" values of MXE events as detected by rMATS in shRNA2 (x-axis) and nt-shRNA (y-axis). Darker colors indicate more significant p-values. Both *Mdm2* MXE events indicated are similar events, defined as separate events by rMATS due to slight differences in the upstream exon.

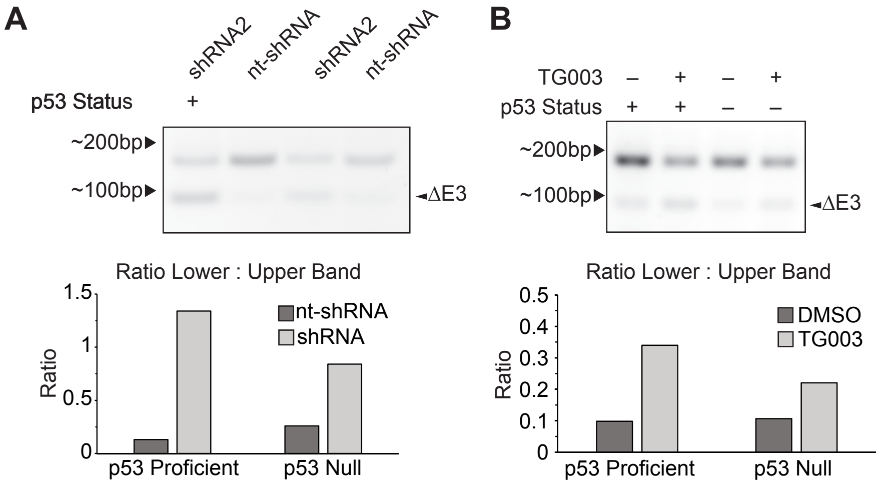

Fig. S3. *Mdm2* alternative splicing in p53-null cells

A. Semi-quantitative Reverse Transcription PCR (sqRT-PCR) of *Mdm2* under *Eftud2* shRNA KD in both p53-proficient and p53-null backgrounds. Quantification of band intensity shown below.
B. sqRT-PCR of *Mdm2* under 24h 100µM TG003 treatment in both p53-proficient and p53-null backgrounds. Quantification of band intensity shown below.

A

	shRNA1		Overlap		shRNA2	
	Up	Down	Up	Down	Up	Down
All Genes	1882		1613		3983	
Alternatively Spliced	45		36		406	
Glycolysis	2		1		4	
Sterol Metabolism	6		4		12	
p53 Transcript. Targets	51		50		118	

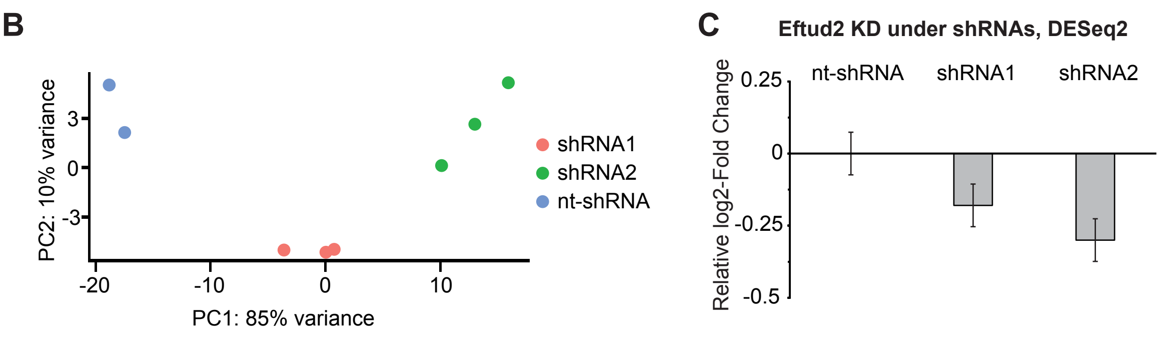

Fig. S4. *Eftud2* KD RNA-Seq summary data

A. Summary of significantly differentially expressed genes (padj < 0.05) under *Eftud2*-targeting shRNAs.
B. Principal Component Analysis of DESeq2 output of *Eftud2* KD and nt-shRNA RNA-Seq
C. Knockdown levels of *Eftud2* as assayed by DESeq2

Disease Models & Mechanisms • Supplementary information

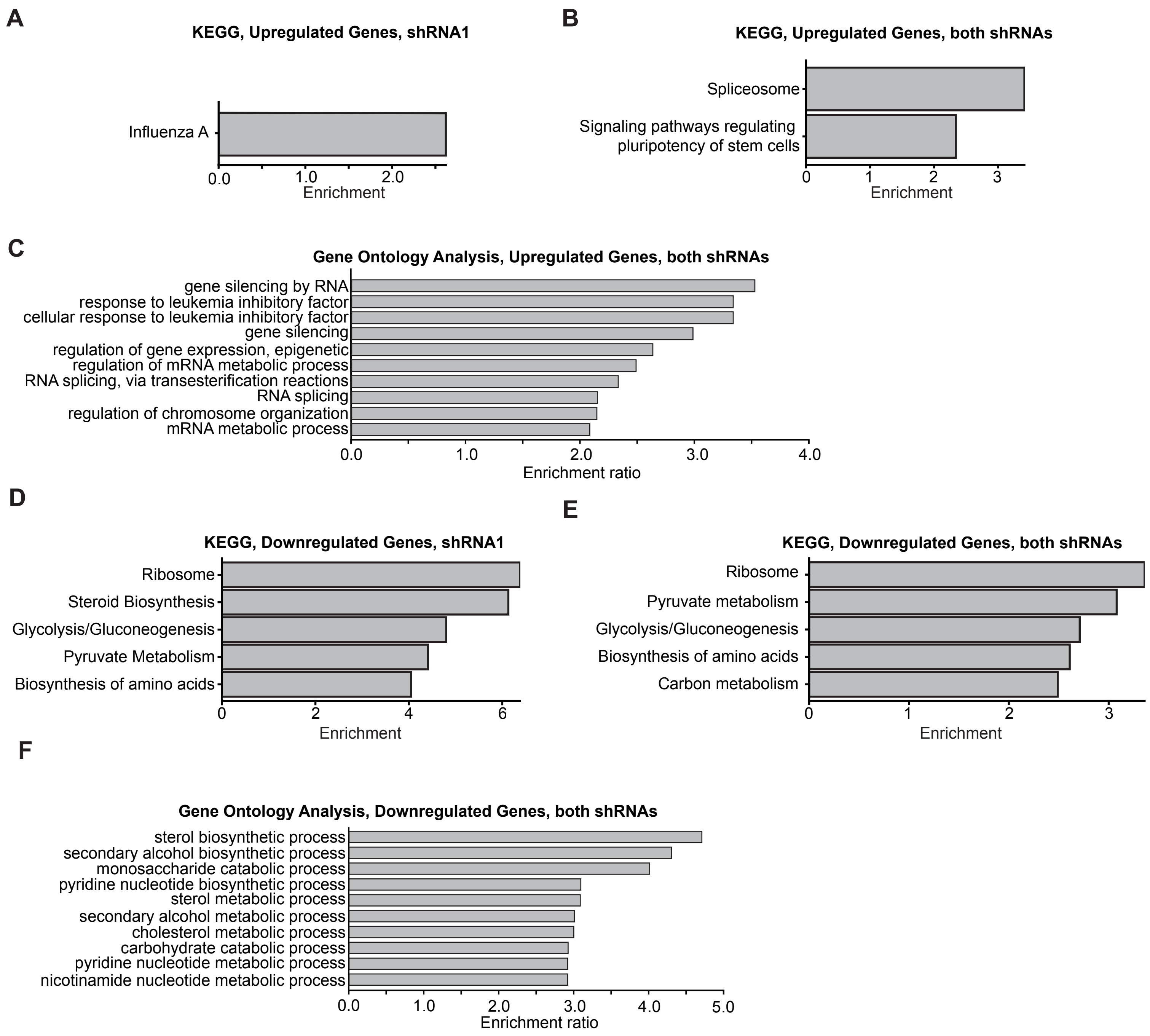

Fig. S5. Additional Gene Ontology and KEGG Over-representation Analysis of *Eftud2* KD RNA-Seq

A. KEGG Pathway over-representation analysis of top 1000 genes most significantly upregulated under shRNA1 compared to nt-shRNA.
B. KEGG Pathway over-representation analysis of all genes commonly significantly upregulated under both *Eftud2*-targeting shRNAs as compared to nt-shRNA
C. Gene Ontology Biological Process over-representation analysis of all commonly significantly upregulated genes under both shRNAs when compared to nt-shRNA.
D. KEGG Pathway analysis of top 1000 genes most significantly downregulated under shRNA1 compared to nt-shRNA.
E. KEGG Pathway analysis of all genes commonly significantly downregulated under both *Eftud2*-targeting shRNAs as compared to nt-shRNA
F. Gene Ontology Biological Process over-representation analysis of all genes commonly significantly downregulated under both shRNAs when compared to nt-shRNA.

Disease Models & Mechanisms • Supplementary information

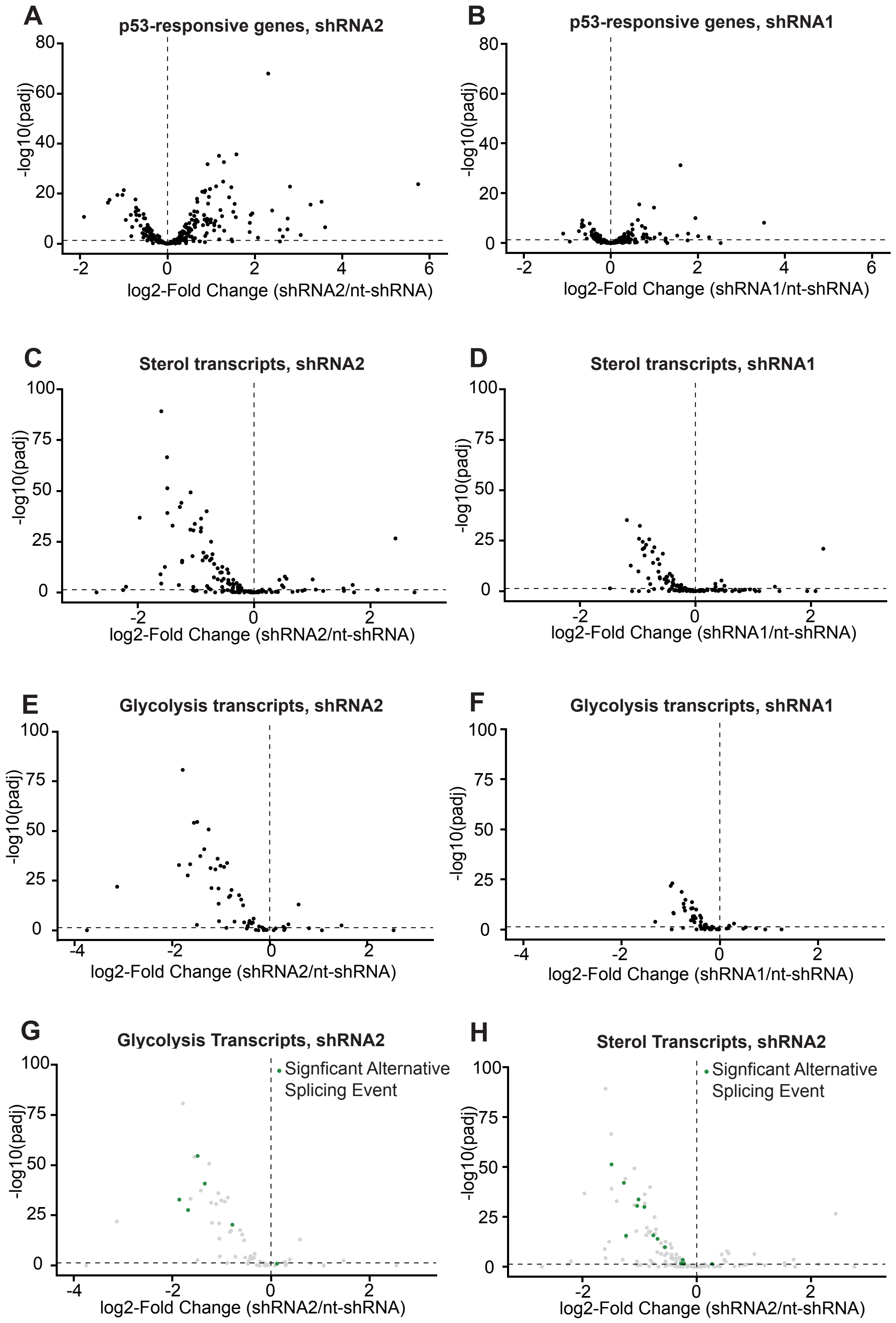

Fig. S6. Volcano plots highlighting gene subsets of *Eftud2* KD DESeq2

A. Differential expression of p53-responsive transcripts in shRNA2, as shown in Fig. 3B
B. Differential expression of p53-responsive transcripts in shRNA1, as shown in Fig. 3C
C. Differential expression of sterol transcripts in shRNA2, as shown in Fig. 3B
D. Differential expression of sterol transcripts in shRNA1, as shown in Fig. 3C
E. Differential expression of glycolysis transcripts in shRNA2, as shown in Fig. 3B
F. Differential expression of glycolysis transcripts in shRNA1, as shown in Fig. 3C
G. Differential expression of glycolysis transcripts under shRNA2 KD, genes with significant alternative splicing events are highlighted in green. There are no glycolytic transcripts significantly alternatively spliced under shRNA1 KD.
H. Differential expression of sterol transcripts under shRNA2 KD, transcripts with significant alternative splicing events are highlighted in green.

Disease Models & Mechanisms • Supplementary information

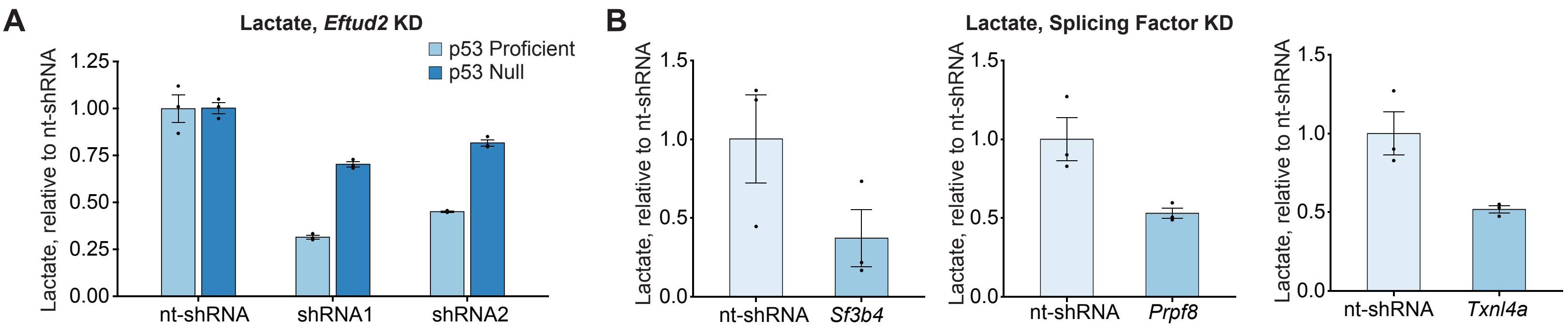

Fig. S7. Lactate Measurements under shRNA knockdown

A. Lactate levels in mESCs treated with the indicated *Eftud2*-targeting shRNA, normalized to lactate levels in nt-shRNA treated mESCs. Lactate readings were standardized to total protein levels. Each point represents a technical replicate, error bars represent standard error of technical replicates; relationship is consistent across multiple biological replicates.

B. Lactate levels in mESCs treated with shRNA targeting the indicated splicing factor, normalized to lactate levels in nt-shRNA treated mESCs. Standardization and error bars as in A).

Disease Models & Mechanisms • Supplementary information

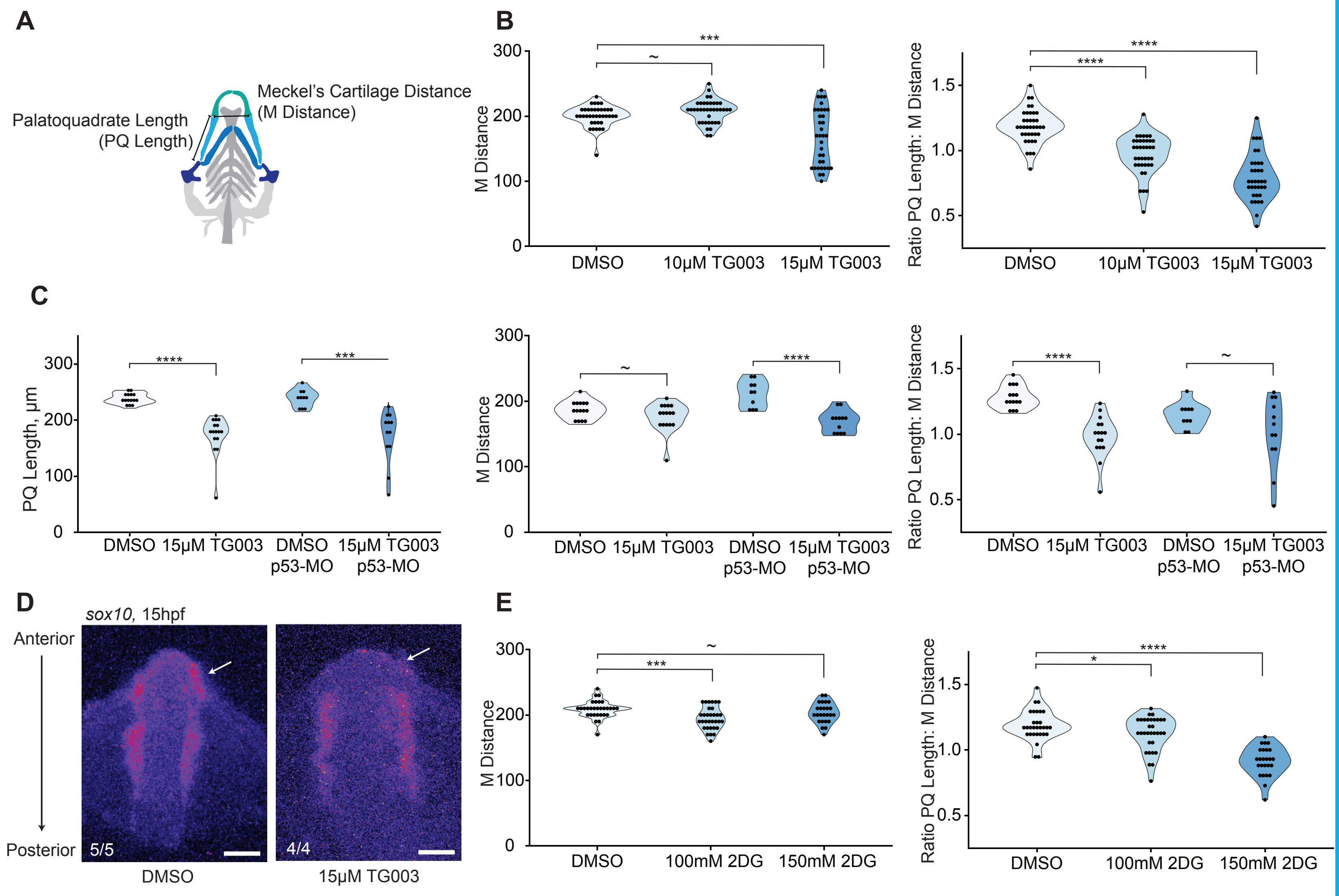

Fig. S8. Additional Characterization of the craniofacial phenotypes in zebrafish

A. Diagram demonstrating additional measurements taken in the 5 dpf zebrafish ventral skeleton. Meckel's Cartilage Distance is used a proxy for width of the craniofacial complex.

B. Measurements and ratios of cartilage lengths in 5 dpf TG003-treated zebrafish and controls. Significance values determined via two-tailed students' t-test. * $p < 0.05$, ** $p < .01$, *** $p < .001$, **** $p < .0001$ ~ p NS.

C. Cartilage measurements and ratios in 5 dpf p53-morphant and control embryos, treated with DMSO or 15µM TG003. Significance and p-value designations as in B)

D. *sox10* expression in 15 hpf embryos treated with DMSO or 15µM TG003, as assayed by HCR. Scale bar represents 100µm. Arrows denote regions of interest in the anterior portion of the embryo.

E. Measurements and ratios of cartilage lengths in 5 dpf 2DG-treated zebrafish and controls. Significance and p-value designations as in B).

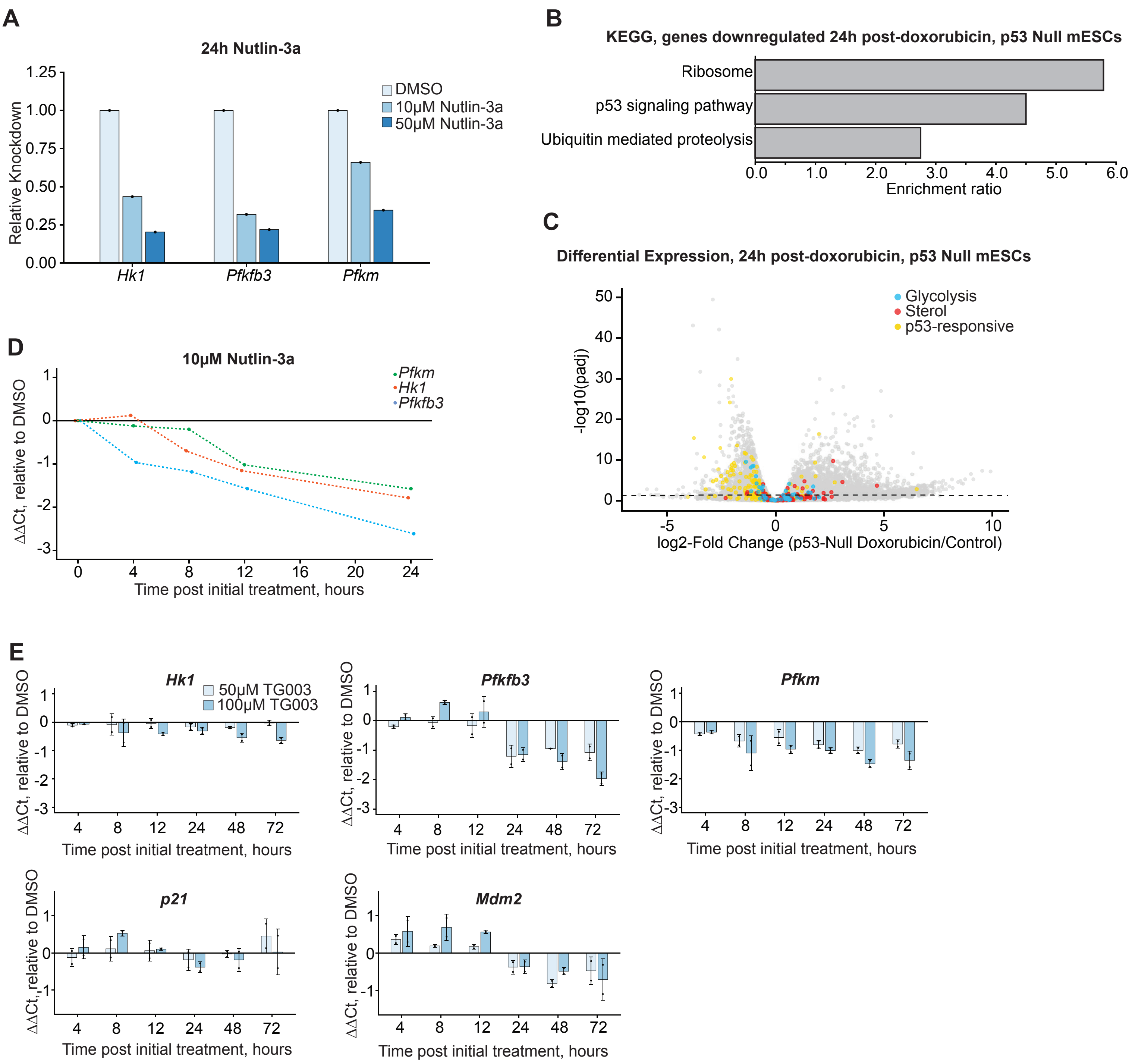

Fig. S9. Further analysis of glycolysis transcripts under p53 stabilizing treatment

A. qPCR of select glycolysis genes in mESCs treated with Nutlin-3a for 24h. ∂Ct values calculated by normalizing raw values to mitochondrial rRNA levels. Plotted values are 2^-∂∂Ct, representing knockdown relative to control DMSO treatment. 1 biological replicate per condition; biological replicate is the average of 3 technical replicates.
B. KEGG Pathway over-representation analysis among genes downregulated under Doxorubicin-treated p53-Null mESCs compared to control-treated mESCs (Grow et al., 2021)
C. Differential expression of all genes comparing Doxorubicin-treated p53 Null mESCs to control-treated mESCs (Grow et al., 2021) as assayed by DESeq2. Glycolysis genes, sterol genes, and p53 transcriptional targets highlighted as in Fig. 3C.
D. qPCR of indicated genes of samples taken at the indicated timepoints. Cells were treated with 10µM Nutlin-3a at 0h. ∂∂Ct values plotted are log2-fold KD compared to control DMSO treatment. ∂Ct values calculated by normalizing raw values to mitochondrial rRNA levels. One biological replicate per condition.
E. qPCR of indicated genes of samples taken at the indicated timepoints. Cells were treated with 50µM or 100µM TG003 at 0h, at 24h (after 24h sample was already collected), and 48h (after 48h sample was already collected). ∂∂Ct values plotted are log-2-fold KD compared to control DMSO treatment. ∂Ct values calculated by normalizing raw values to mitochondrial rRNA levels. Two biological replicates per condition, each biological replicate is the average of three technical replicates. Error bars represent standard deviation of the biological replicates.

Table S1. Primers Used

Primer Target	Purpose	Organism	Forward Primer (5-->3)	Reverse Primer (5-->3)
mt 16s rRNA ref	qPCR	Mm	CCGCAAGGGAAAGATGAAAGAC	TCGTTTGGTTTCGGGGTTTC
Hk1	qPCR	Mm	ACTGGATCTCGGCGGGTCTTCC	GCGACGTGATCAAAAAGCTGGC
Pfkfb3	qPCR	Mm	GTGGGCCGAAGCTGACCAACTC	GGGCAGCTAAGGCACACTGTTTT
Pfkm	qPCR	Mm	TCCATGAGGGTTACCAAGGCCTGG	TGATCCCCCGCTTCACCAGGTT
p21	qPCR	Mm	CCTGGTGATGTCCGACCTGTTC	CCATGAGCGCATCGCAATCACG
Mdm2	qPCR	Mm	CCAACCATCGACTTCCAGCAGCATT	GATTGGCTGTCTGCACACTGGG
Mdm2 Exons 2-4	sqRT-PCR	Mm	GATCACCGCGCTTCTCCTGC	TGTCGTTTTGCGCTCCAACGGA
p53	sgRNA	Mm	CACCGCCTCGAGCTCCCTCTGAGCC	AAACGGCTCAGAGGGAGCTCGAGGC
mtRNA ND3-ND4L ref	qPCR	Dr	CCTACGAATGAGCCCAAGG	CGGTGAAATGTAAGTCCTGCT
Gapdh	qPCR	Dr	GTGGAGTCTACTGGTGTCTTC	GTGCAGGAGGCATTGCTTACA
Pfklb	qPCR	Dr	CGACAGGAACTTCGGCACTA	CCAATCACACATGCCGTATC
Hk1	qPCR	Dr	CTGCAATTGAGAAGAGCAAGGA	GTTCTTGTTGTCCTTCAGACGT
Eno1a	qPCR	Dr	GTTATCAACGGCGGCTCTCATG	GCTCCAGAGCTTCTTTGTTCTCA
Mdm2 Exons 2-5	sqRT-PCR	Dr	CAGTTCTCAGATCAGCAAGG	GTTGATCAATGCAAAGAGGGC

Disease Models & Mechanisms • Supplementary information

Table S2. qPCR data

∂Ct values of qPCR data, normalized to mitochondrial rRNA unless otherwise stated Unless otherwise stated, 3 technical replicates, SE = sqrt((SDmito/sqrt(3))^2 + (SDtarget/sqrt(3))^2)

Fig. 1A: Eftud2 KD under shRNA treatment

	Eftud2 Prime	SE	Eftud2 Prime	SE
shRNA1-A	-10.718333	0.01043055	-12.035	0.01736056
shRNA1-B	-11.106667	0.06842135	-12.463333	0.05818234
shRNA1-C	-10.656667	0.05682983	-12.383333	0.29995061
shRNA2-A	-11.476667	0.02018434	-13.053333	0.21173009
shRNA2-B	-11.27	0.07845735	-12.67	0.0294392
shRNA2-C	-11.016667	0.00981307	-12.493333	0.03079201
nt-shRNA-A	-9.5566667	0.00860663	-10.766667	0.01186342
nt-shRNA-B	-9.55	0.01677741	-10.843333	0.04286067
nt-shRNA-C	-9.3833333	0.0261052	-10.62	0.03220306

Fig. 3F. Eftud2 shRNA, p53 proficient & null

	Eftud2 avg	Eftud2 SE	Hk1 avg	Hk1 SE	Pfkfb3 avg	Pfkfb3 SE	Pfkm avg	Pfkm SE
shRNA1-A	-9.6766667	0.04136558	-10.723333	0.04876246	-13.91	0.05715476	-12.22	0.0391578
shRNA1-B	-9.9933333	0.06863753	-10.873333	0.01763834	-14.096667	0.04666667	-12.38	0.02708013
shRNA1-C	-11.11	0.01885618	-12.256667	0.01333333	-14.1	0.01699673	-14.316667	0.03887301
shRNA2-A	-8.48	0.03366502	-10.503333	0.01763834	-12.936667	0.05333333	-11.88	0.02309401
shRNA2-C	-10.723333	0.07063207	-11.946667	0.08628119	-13.673333	0.06699917	-14.176667	0.07241854
NTC-A	-6.68	0.06497863	-7.7633333	0.06236096	-11.11	0.06798693	-9.97	0.06289321
NTC-B	-6.9433333	0.04055175	-7.8133333	0.07007932	-11.236667	0.03711843	-10.096667	0.04876246
NTC-C	-8.8366667	0.02538591	-8.7633333	0.03231787	-10.63	0.03511885	-11.853333	0.03382964
p53 Null shRN	-9.7133333	0.04521553	-10.066667	0.01855921	-12.666667	0.07102425	-12.106667	0.01855921
p53 Null shRN	-9.98	0.08768631	-10.463333	0.04371626	-12.773333	0.08472177	-12.456667	0.06749486
p53 Null shRN	-11.113333	0.03248931	-12.263333	0.03036811	-13.526667	0.03179797	-14.636667	0.04944132
p53 NullshRN	-9.6966667	0.03844188	-10.236667	0.02905933	-12.313333	0.03197221	-12.216667	0.07731609
p53 NullshRN	-9.91	0.04714045	-10.473333	0.07264832	-12.663333	0.0421637	-12.573333	0.04737557
p53 NullshRN	-11.126667	0.02788867	-12.4	0.02581989	-13.36	0.05228129	-14.59	0.04281744
p53 Null NTC	-7.1366667	0.03382964	-9.1966667	0.01763834	-11.25	0.02211083	-11.166667	0.01763834
p53 Null NTC	-7.0433333	0.04594683	-8.83	0.03349959	-11.076667	0.03349959	-10.843333	0.03431877
p53 Null NTC	-8.72	0.02426703	-10.82	0.02134375	-12.63	0.02867442	-12.953333	0.01795055

Fig. 4A. Prpf8 shRNA

	Prpf8 avg	Prpf8 SE	Hk1 avg	Hk1 SE	Pfkfb3 avg	Pfkfb3 SE	Pfkm avg	Pfkm SE
shRNA - A	-8.2733333	0.02905933	-10.283333	0.01563472	-13.836667	0.01333333	-12.483333	0.00881917
shRNA - B	-8.2066667	0.00333333	-10.21	0.01	-13.77	0.01	-12.47	0.03785939
shRNA - C	-16.636667	0.27944389	-9.53	0.04268749	-13.333333	0.02027588	-12.123333	0.42333333
shRNA - D	-16.37	0.16489054	-9.1666667	0.01333333	-13.286667	0.04558265	-11.983333	0.49486249
NTC - A			-8.9566667	0.01333333	-11.086667	0.02260777	-11.336667	0.02472066
NTC - B	-7.9566667	0.01763834	-8.9233333	0.02333333	-11.963333	0.02027588	-11.443333	0.02403701
NTC - C	-14.873333	0.25766041	-7.7633333	0.06236096	-11.11	0.06798693	-9.97	0.06289321
NTC - D	-15.24	0.25197002	-7.8133333	0.07007932	-11.236667	0.03711843	-10.096667	0.04876246

Fig. 4A. Txnl4a shRNA

	Txnla4a avg	Txnl4a SE	Hk1 avg	Hk1 SE	Pfkfb3 avg	Pfkfb3 SE	Pfkm avg	Pfkm SE
shRNA - A	-16.416667	0.14586904	-10.276667	0.00881917	-14.04	0.0765216	-12.7	0.02285218
shRNA - B	-15.97	0.1785746	-10.01	0.02054805	-13.686667	0.06896054	-12.353333	0.02185813
shRNA - C	-13.563333	0.20113566	-8.9966667	0.02905933	-12.003333	0.04268749	-11.74	0.2038518
shRNA - D	-14.273333	0.08724168	-8.96	0.07866949	-12.316667	0.06699917	-12.033333	0.39838563
NTC - A	-14.28	0.01290994	-8.9566667	0.01333333	-11.086667	0.02260777	-11.336667	0.02472066
NTC - B			-8.9233333	0.02333333	-11.963333	0.02027588	-11.443333	0.02403701
NTC - C	-11.303333	0.08730534	-7.7633333	0.06236096	-11.11	0.06798693	-9.97	0.06289321
NTC - D	-11.74	0.01490712	-7.8133333	0.07007932	-11.236667	0.03711843	-10.096667	0.04876246

Fig 4A,B. Sf3b4 shRNA

Sf3b4 shRNA	Sf3b4 avg	Sf3b4 SE	Hk1 avg	Hk1 SE	Pfkfb3 avg	Pfkfb3 SE	Pfkm avg	Pfkm SE
shRNA-A	-7.7733333	0.03073181	-10.243333	0.02108185	-12.916667	0.01943651	-11.936667	0.02108185

shRNA-B	-8.83	0.03299832	-10.546667	0.03496029	-13.81	0.10561986	-12.43	0.07039571
shRNA-C	-11.663333	0.02472066	-10.046667	0.02134375	-12.956667	0.01972027	-12.96	0.03349959
shRNA-D	-11.273333	0.02981424	-10.606667	0.03844188	-13.486667	0.03382964	-13.18	0.0314466
NTC-A	-6.3266667	0.06280481	-7.7633333	0.06236096	-11.11	0.06798693	-9.97	0.06289321
NTC-B	-6.65	0.03651484	-7.8133333	0.07007932	-11.236667	0.03711843	-10.096667	0.04876246
NTC-C	-10.11	0.01972027	-9.15	0.02924988	-11.28	0.02134375	-11.59	0.01374369
NTC-D			-8.9566667	0.01333333	-11.086667	0.02260777	-11.336667	0.02472066
p53 Null shRN	-8.9833333	0.04977728	-9.7366667	0.04136558	-12.606667	0.06936217	-12.126667	0.02848001
p53 Null shRN	-9.1566667	0.03333333	-9.9633333	0.02788867	-12.666667	0.03527668	-12.363333	0.05011099
p53 Null shRN	-11.233333	0.04176655	-9.6	0.04203173	-12.553333	0.04333333	-12.666667	0.04807402
p53 Null NTC-	-7.1166667	0.0617342	-9.1966667	0.01763834	-11.25	0.02211083	-11.166667	0.01763834
p53 Null NTC-	-7.1133333	0.03480102	-8.83	0.03349959	-11.076667	0.03349959	-10.843333	0.03431877
p53 Null NTC	-9.7633333	0.00666667	-9.6033333	0.00666667	-12.923333	0.00333333	-12.136667	0.01795055

Fig 4C,D: 100µM TG003

	Hk1 avg	Hk1 SE	Pfkfb3 avg	Pfkfb3 SE	Pfkm avg	Pfkm SE
DMSO - A	-11.04	0.04384315	-12.4	0.04749269	-13.01	0.04533824
DMSO - B	-10.95	0.02211083	-11.96	0.02867442	-12.883333	0.02624669
TG - A	-12.943333	0.03711843	-14.946667	0.03496029	-14.653333	0.03431877
TG - B	-12.89	0.03771236	-14.543333	0.01490712	-14.443333	0.03091206
p53 Null DMS	-10.996667	0.02403701	-12.11	0.02516611	-12.96	0.0728011
p53 Null DMS	-11.003333	0.01054093	-12.053333	0.01763834	-12.943333	0.02027588
p53 Null TG -	-11.453333	0.03929942	-13.97	0.03651484	-13.45	0.03651484
p53 Null TG -	-11.403333	0.03126944	-13.89	0.02624669	-13.416667	0.02981424

Fig 5D: 24h bulk zebrafish extracts, normalized to mtRNA ND3-ND4L

	Gapdh	Gapdh SE	Hk1	Hk1 SE	Pfkl	Pfkl SE	Eno1a	Eno1a SE
DMSO	1.74	0.09955456	-3.9266667	0.11411495	2.01333333	0.08339997	0.91333333	0.08975275
10µM TG003	-0.7266667	0.08956686	-5.72	0.09786612	-0.5433333	0.07542472	-1.8266667	0.1076517
15µM TG003	-1.4166667	0.12644718	-5.8433333	0.12306277	-0.35	0.10044346	-1.8766667	0.11657139
p53-MO DMS	0.08666667	0.08844333	-5.7333333	0.08825468	-0.6133333	0.08673075	-1.8766667	0.1180866
p53-MO 10µM	-1.7033333	0.06218253	-5.56	0.05783117	1.04333333	0.04702245	-0.8233333	0.06608076
p53-MO 15µM	-1.9433333	0.16182981	-5.9866667	0.21944374	-0.49	0.07401201	-2.5766667	0.1117537

Fig 6A: Doxorubicin Concentration Gradient

	Hk1	Hk1 SE	Pfkfb3	Pfkfb3 SE	Pfkm	Pfkm SE
DMSO	-9.0433333	0.06306963	-11.206667	0.05962848	-11.36	0.05374838
50nM Doxo	-10.456667	0.0664162	-13.79	0.02236068	-11.963333	0.03073181
200nM Doxo	-11.37	0.03741657	-14.72	0.06271629	-12.806667	0.03231787
1µM Doxo	-13.256667	0.05487359	-15.526667	0.08130874	-14.73	0.1132843

Fig 6B: 200nM Doxorubicin - p53 proficient & null

	Hk1 avg	Hk1 SE	Pfkfb3 avg	Pfkfb3 SE	Pfkm avg	Pfkm SE
DMSO - A	-10.09	0.0305505	-13.87	0.02828427	-12.403333	0.09333333
DMSO - B	-10.043333	0.01105542	-13.89	0.01885618	-12.436667	0.13678856
DMSO - C	-10.67	0.00745356	-12.946667	0.02426703	-12.926667	0.00942809
Doxo - A	-11.913333	0.03179797	-15.18	0.03958114	-14.12	0.10939226
Doxo - B	-12.19	0.03162278	-15.656667	0.05577734	-14.356667	0.0421637
Doxo - C	-12.936667	0.00942809	-15.453333	0.0120185	-14.263333	0.02666667
p53 Null DMS	-10.043333	0.01943651	-13.126667	0.01490712	-12.526667	0.05497474
p53 Null DMS	-9.62	0.06209312	-12.646667	0.06749486	-12.06	0.0837987
p53 Null DMS	-14.316667	0.07688375	-9.9833333	0.02494438	-13.086667	0.34031032
p53 Null Doxo	-10.546667	0.01054093	-12.453333	0.00942809	-12.31	0.03944053
p53 Null Doxo	-10.436667	0.05109903	-12.413333	0.05426274	-12.273333	0.06666667
p53 Null Doxo	-15.23	0.04678556	-10.733333	0.01563472	-12.92	0.02054805

Fig 6E: Doxorubicin timecourse

	Hk1	Hk1 SE	Pfkfb3	Pfkfb3 SE	Pfkm	Pfkm SE	p21	p21 SE	Mdm2	Mdm2 SE
4h DMSO A	-9.5566667	0.02	-13.36	0.04281744	-11.833333	0.02309401	-10.933333	0.04055175	-7.9466667	0.04521553
4h DMSO B	-10.366667	0.02108185	-14.2	0.06608076	-12.103333	0.03711843	-11.73	0.19407902	-8.47	0.02624669

4h Doxo	-9.7433333	0.0601849	-14.356667	0.07774603	-11.72	0.06128259	-9.95	0.06674995	-6.64	0.03448027
4h Doxo	-10.276667	0.02357023	-14.32	0.11352924	-12.053333	0.01763834	-10.993333	0.05696002	-6.41	0.02380476
8h DMSO A	-9.2066667	0.02185813	-13.983333	0.10624918	-11.166667	0.04307616	-11.183333	0.04853407	-8.0983333	0.05222813
8h DMSO B	-9.9133333	0.01855921	-14.683333	0.08252946	-11.86	0.0321455	-11.69	0.08582929	-8.7833333	0.0120185
8h Doxo A	-9.9366667	0.04594683	-14.276667	0.05868939	-11.393333	0.04521553	-10.913333	0.08705043	-6.8066667	0.05436502
8h Doxo B	-10.726667	0.02108185	-15.073333	0.10697871	-12.163333	0.03972125	-11.59	0.10311806	-7.0433333	0.01054093
12h DMSO A	-9.48	0.03887301	-14.213333	0.08589399	-11.373333	0.0421637	-11.22	0.06765928	-8.1633333	0.02560382
12h DMSO B	-9.64	0.02309401	-14.573333	0.02260777	-11.72	0.01414214	-11.486667	0.06253888	-8.4866667	0.03018462
12h Doxo A	-10.096667	0.04136558	-14.753333	0.04876246	-11.23	0.05163978	-10.953333	0.07055337	-6.6466667	0.03480102
12h Doxo B	-10.29	0.02768875	-14.593333	0.05953524	-11.593333	0.04013865	-11.773333	0.06790516	-6.4066667	0.01885618
24h DMSO A	-8.5933333	0.02924988	-12.443333	0.03527668	-10.79	0.02788867	-10.943333	0.07272475	-7.8333333	0.1226558
24h DMSO B	-8.2866667	0.00942809	-12.446667	0.05557777	-10.366667	0.02357023	-10.483333	0.05044249	-7.7766667	0.05011099
24h Doxo A	-10.686667	0.04807402	-15.236667	0.08252946	-12.083333	0.05647025	-12.053333	0.04955356	-7.9233333	0.03431877
24h Doxo B	-11.276667	0.04853407	-15.35	0.05374838	-13.033333	0.02666667	-12.57	0.0372678	-7.7566667	0.03431877

Fig 6F: TG003 timecourse

	Hk1	Hk1 SE	Pfkfb3	Pfkfb3 SE	Pfkm	Pfkm SE	p21	p21 SE	Mdm2	Mdm2 SE
4h DMSO A	-9.5566667	0.02	-13.36	0.04281744	-11.833333	0.02309401	-10.933333	0.04055175	-8.47	0.02624669
4h DMSO B	-10.216667	0.03382964	-13.61	0.06683313	-12.293333	0.08232726	-12.243333	0.07241854	-8.82	0.0314466
4h 50 TG A	-9.62	0.02687419	-13.52	0.03036811	-12.23	0.01490712	-10.883333	0.02924988	-8.0066667	0.07695598
4h 50 TG B	-10.366667	0.02027588	-13.86	0.10214369	-12.753333	0.04630815	-12.543333	0.04666667	-8.5466667	0.02788867
4h 100 TG A	-9.63	0.02333333	-13.336667	0.0760117	-12.24	0.02185813	-10.566667	0.12292726	-7.6	0.04027682
4h 100 TG B	-10.283333	0.01563472	-13.413333	0.06119187	-12.613333	0.08894443	-12.31	0.06236096	-8.52	0.01154701
8h DMSO A	-9.2066667	0.02185813	-13.983333	0.10624918	-11.166667	0.04307616	-11.183333	0.04853407	-8.1266667	0.04988877
8h DMSO B	-10.45	0.01490712	-14.406667	0.04176655	-12.283333	0.01247219	-12.763333	0.1667	-8.7733333	0.03901567
8h 50 TG A	-9.5533333	0.04027682	-14.18	0.03496029	-11.99	0.02905933	-11.306667	0.02357023	-7.96	0.04384315
8h 50 TG B	-10.273333	0.02211083	-14.326667	0.05142416	-12.806667	0.02538591	-12.423333	0.06368324	-8.5566667	0.04447221
8h 100 TG A	-9.93	0.02309401	-13.31	0.02108185	-12.693333	0.03620927	-10.6	0.04690416	-7.1866667	0.08647415
8h 100 TG B	-10.476667	0.02426703	-13.836667	0.15315207	-12.953333	0.03666667	-12.29	0.08319989	-8.33	0.03036811
12h DMSO A	-9.48	0.03887301	-14.213333	0.08589399	-11.373333	0.0421637	-11.22	0.06765928	-8.17	0.04582576
12h DMSO B	-9.92	0.01527525	-14.153333	0.02333333	-11.823333	0.01666667	-12.163333	0.04841946	-8.6833333	0.01333333
12h 50 TG A	-9.4033333	0.01490712	-14.093333	0.06674995	-11.726667	0.01885618	-10.956667	0.02357023	-7.95	0.02426703
12h 50 TG B	-10.076667	0.01763834	-14.61	0.05131601	-12.57	0.03464102	-12.303333	0.1537675	-8.5666667	0.03231787
12h 100 TG A	-9.8533333	0.03036811	-13.55	0.04818944	-12.433333	0.02560382	-11.1	0.05249339	-7.58	0.04533824
12h 100 TG B	-10.383333	0.04749269	-14.223333	0.06624869	-12.683333	0.02054805	-12.086667	0.06863753	-8.1433333	0.02027588
24h DMSO A	-8.5933333	0.02924988	-12.443333	0.03527668	-10.79	0.02788867	-10.943333	0.07272475	-7.91	0.03496029
24h DMSO B	-9.3933333	0.02687419	-12.296667	0.03756476	-11.323333	0.02808717	-12.686667	0.14970341	-8.97	0.01885618
24h 50 TG A	-8.8566667	0.01105542	-13.923333	0.02185813	-11.493333	0.02472066	-11.336667	0.06565905	-8.41	0.0305505
24h 50 TG B	-9.4766667	0.04333333	-13.233333	0.05792716	-12.233333	0.05217492	-12.67	0.05557777	-9.2133333	0.02472066
24h 100 TG A	-8.9966667	0	-13.433333	0	-11.733333	0	-11.433333	0	-8.1433333	0.04384315
24h 100 TG B	-9.6166667	0.02108185	-13.613333	0.03248931	-12.4	0.02808717	-12.963333	0.09893881	-9.4633333	0.02494438
48h DMSO A	-8.85	0.04988877	-12.073333	0.04876246	-10.443333	0.04944132	-11.663333	0.08412953	-8.12	0.03944053
48h DMSO B	-9.83	0.01247219	-12.003333	0.01763834	-11.31	0.02867442	-13.303333	0.02538591	-9.4933333	0.01452966
48h 50 TG A	-9.0666667	0.03800585	-13.023333	0.04013865	-11.36	0.04163332	-11.763333	0.08628119	-9.0066667	0.04459696
48h 50 TG B	-9.99	0.01105542	-12.95	0.02054805	-12.396667	0.02666667	-13.263333	0.05849976	-10.233333	0.04921608
48h 100 TG A	-9.2833333	0.03382964	-13.26	0.03829708	-11.816667	0.03527668	-11.63	0.05507571	-8.6666667	0.06101002
48h 100 TG B	-10.493333	0.01943651	-13.596667	0.01666667	-12.89	0.02768875	-13.713333	0.03231787	-9.9066667	0.04841946
72h DMSO A	-9.1066667	0.04484541	-11.57	0.04496913	-10.466667	0.04594683	-11.846667	0.06009252	-8.31	0.03986087
72h DMSO B	-10.04	0.00942809	-11.75	0.00745356	-11.483333	0.02054805	-13.576667	0.02666667	-9.74	0.04966555
72h 50 TG A	-9.2033333	0.05527708	-12.85	0.04496913	-11.346667	0.04876246	-11.72	0.06315765	-9.0433333	0.03333333
72h 50 TG B	-9.9966667	0.01855921	-12.633333	0.0120185	-12.163333	0.02185813	-12.8	0.03785939	-9.95	0.01914854
72h 100 TG A	-9.6666667	0.02538591	-13.376667	0.02027588	-11.583333	0.01763834	-11.386667	0.02108185	-8.6166667	0.02728451
72h 100 TG B	-10.756667	0.01374369	-13.88	0.01105542	-13.073333	0.00666667	-13.983333	0.04096069	-10.833333	0.01699673

Fig. S9A. Nutlin-3a Concentration Gradient

	Hk1	Hk1 SE	Pfkfb3	Pfkfb3 SE	Pfkm	Pfkm SE
DMSO	-9.0433333	0.06306963	-11.206667	0.05962848	-11.36	0.05374838
10µM Nut	-10.243333	0.04818944	-12.86	0.04988877	-11.956667	0.0788106
50µM Nut	-11.34	0.06749486	-13.396667	0.06280481	-12.893333	0.06182412

Fig. S9D. 10µM Nutlin-3a timecourse

	Hk1	Hk1 SE	Pfkfb3	Pfkfb3 SE	Pfkm	Pfkm SE
4h DMSO	-10.746667	0.02666667	-14.886667	0.05142416	-12.703333	0.01795055
4h Nut	-10.623333	0.01795055	-15.853333	0.0745356	-12.823333	0.04229526
8h DMSO	-10.78	0.02211083	-14.97	0.05792716	-12.746667	0.03197221
8h Nut	-11.476667	0.03018462	-16.153333	0.10780641	-12.946667	0.02260777
12h DMSO	-10.403333	0.01598611	-14.7	0.05249339	-12.333333	0.01699673
12h Nut	-11.556667	0.02538591	-16.266667	0.07774603	-13.356667	0.01763834
24h DMSO	-10.163333	0.03496029	-13.866667	0.05577734	-12.083333	0.04496913
24h Nut	-11.94	0.03109126	-16.476667	0.14869805	-13.653333	0.01943651

Table S3. DESeq2 output

Available for download at
https://journals.biologists.com/dmm/article-lookup/doi/10.1242/dmm.050356#supplementary-data

Table S4. rMATS filtered output

Available for download at
https://journals.biologists.com/dmm/article-lookup/doi/10.1242/dmm.050356#supplementary-data

Supplementary References

Bowen, M.E., McClendon, J., Long, H.K., Sorayya, A., Van Nostrand, J.L., Wysocka, J., Attardi, L.D., 2019. The Spatiotemporal Pattern and Intensity of p53 Activation Dictates Phenotypic Diversity in p53-Driven Developmental Syndromes. Dev. Cell 50, 212-228.e6. https://doi.org/10.1016/j.devcel.2019.05.015

Dingerkus, G., Uhler, L.D., 1977. Enzyme clearing of alcian blue stained whole small vertebrates for demonstration of cartilage. Stain Technol. 52, 229–232. https://doi.org/10.3109/10520297709116780

Dobin, A., Davis, C.A., Schlesinger, F., Drenkow, J., Zaleski, C., Jha, S., Batut, P., Chaisson, M., Gingeras, T.R., 2013. STAR: ultrafast universal RNA-seq aligner. Bioinformatics 29, 15–21. https://doi.org/10.1093/bioinformatics/bts635

Grow, E.J., Weaver, B.D., Smith, C.M., Guo, J., Stein, P., Shadle, S.C., Hendrickson, P.G., Johnson, N.E., Butterfield, R.J., Menafra, R., Kloet, S.L., van der Maarel, S.M., Williams, C.J., Cairns, B.R., 2021. p53 convergently activates Dux/DUX4 in embryonic stem cells and in facioscapulohumeral muscular dystrophy cell models. Nat. Genet. 53, 1207–1220. https://doi.org/10.1038/s41588-021-00893-0

Lee, K.-H., Li, M., Michalowski, A.M., Zhang, X., Liao, H., Chen, L., Xu, Y., Wu, X., Huang, J., 2010. A genomewide study identifies the Wnt signaling pathway as a major target of p53 in murine embryonic stem cells. Proc. Natl. Acad. Sci. U. S. A. 107, 69–74. https://doi.org/10.1073/pnas.0909734107

Li, M., He, Y., Dubois, W., Wu, X., Shi, J., Huang, J., 2012. Distinct regulatory mechanisms and functions of p53-activated and p53-repressed DNA damage response genes in embryonic stem cells. Mol. Cell 46, 30–42. https://doi.org/10.1016/j.molcel.2012.01.020

Liao, Y., Smyth, G.K., Shi, W., 2014. featureCounts: an efficient general purpose program for assigning sequence reads to genomic features. Bioinformatics 30, 923–930. https://doi.org/10.1093/bioinformatics/btt656

Liao, Y., Wang, J., Jaehnig, E.J., Shi, Z., Zhang, B., 2019. WebGestalt 2019: gene set analysis toolkit with revamped UIs and APIs. Nucleic Acids Res. 47, W199–W205. https://doi.org/10.1093/nar/gkz401

Love, M.I., Huber, W., Anders, S., 2014. Moderated estimation of fold change and dispersion for RNA-seq data with DESeq2. Genome Biol. 15, 550. https://doi.org/10.1186/s13059-014-0550-8

Sakata-Haga, H., Uchishiba, M., Shimada, H., Tsukada, T., Mitani, M., Arikawa, T., Shoji, H., Hatta, T., 2018. A rapid and nondestructive protocol for whole-mount bone staining of small fish and Xenopus. Sci. Rep. 8, 7453. https://doi.org/10.1038/s41598-018-25836-4

Shen, S., Park, J.W., Lu, Z., Lin, L., Henry, M.D., Wu, Y.N., Zhou, Q., Xing, Y., 2014. rMATS: Robust and flexible detection of differential alternative splicing from replicate RNA-Seq data. Proc. Natl. Acad. Sci. 111, E5593–E5601. https://doi.org/10.1073/pnas.1419161111

Thomson, M., Liu, S.J., Zou, L.-N., Smith, Z., Meissner, A., Ramanathan, S., 2011. Pluripotency Factors in Embryonic Stem Cells Regulate Differentiation into Germ Layers. Cell 145, 875–889. https://doi.org/10.1016/j.cell.2011.05.017

Ying, Q.-L., Smith, A.G., 2003. Defined Conditions for Neural Commitment and Differentiation, in: Methods in Enzymology, Differentiation of Embryonic Stem Cells. Academic Press, pp. 327–341. https://doi.org/10.1016/S0076-6879(03)65023-8

Ying, Q.-L., Wray, J., Nichols, J., Batlle-Morera, L., Doble, B., Woodgett, J., Cohen, P., Smith, A., 2008. The ground state of embryonic stem cell self-renewal. Nature 453, 519–523. https://doi.org/10.1038/nature06968

Disease Models & Mechanisms • Supplementary information
